# EKEO: An Enhanced Kangaroo Escape Optimizer with Balanced Search for Global Optimization and Engineering Design

**DOI:** 10.3390/biomimetics11050308

**Published:** 2026-05-01

**Authors:** Xuemei Zhu, Weijie Guo, Yang Shen, Jingchun Guo, Shirong Li, Zhiqiang Chang

**Affiliations:** 1 Experimental and Practical Training Teaching Management Department, West Anhui University, Lu’an 237012, China; 14000012@wxc.edu.cn (X.Z.); 43000013@wxc.edu.cn (J.G.); 2 School of Electrical and Optoelectronic Engineering, West Anhui University, Lu’an 237012, China; 2023010521@mail.wxc.edu.cn (W.G.); 2023010489@mail.wxc.edu.cn (Y.S.); 3 Automotive Technology College, Anhui Vocational College of Defense Technology, Lu’an 237011, China; 35311485@acdt.edu.cn; 4 School of Electronic Information and Artificial Intelligence, West Anhui University, Lu’an 237012, China

**Keywords:** kangaroo escape optimizer, differential evolution mutation, quasi-oppositional learning, engineering optimization, metaheuristic algorithms

## Abstract

The Kangaroo Escape Optimizer (KEO) is a recently proposed biomimetic metaheuristic inspired by the adaptive escape strategies of kangaroos in predator–prey interactions. Although effective, KEO-like algorithms based on many populations may suffer from premature convergence and loss of population diversity when addressing complex, multimodal, and constrained optimization problems. This paper proposes an Enhanced Kangaroo Escape Optimizer (EKEO) that integrates Differential Evolution Mutation (DEM) and Quasi-Oppositional Learning (QOL) to address fundamental limitations in exploration–exploitation balance. From a biomimetic perspective, DEM mimics the refined high-frequency muscular adjustments of a kangaroo during close-range evasion, enabling local refinement around promising solutions, while QOL emulates the animal’s sudden directional changes and scanning behavior to preserve population diversity and escape local optima. Their principled integration yields a robust optimization framework that consistently outperforms state-of-the-art and classical metaheuristics across benchmark functions and real-world engineering problems. The findings suggest a generalizable design principle for biomimetic hybrid metaheuristics, demonstrating that coupling directed exploitation with diversity-preserving exploration leads to reliable high-performance optimization. The performance of EKEO is rigorously evaluated in two phases. First, its optimization accuracy and convergence speed are benchmarked against 11 state-of-the-art and classical metaheuristics on 23 classical benchmark functions and the CEC 2019 test suite. Second, its practical applicability and constraint-handling effectiveness are validated on four real-world engineering design problems: step-cone pulley, gear system, tubular column, and pressure vessel design. The experimental results are supported by comprehensive statistical analyses (including Wilcoxon rank-sum tests) and convergence curves, showing that EKEO consistently outperforms its competitors in solution quality, convergence speed, and robustness. These findings establish EKEO as a competitive, reliable, and versatile biomimetic optimization tool suitable for solving complex continuous and constrained engineering optimization problems.

## 1. Introduction

Optimization problems pervade nearly every field of science and engineering, from structural design and control systems to resource allocation and machine learning [1]. As real-world problems grow increasingly complex, involving nonlinear relationships, mixed variables, multiple constraints, and high-dimensional search spaces, the demand for efficient and robust optimization algorithms has continued to intensify [2]. Traditional gradient-based methods often struggle with such complexities due to their reliance on derivative information and sensitivity to initial guesses [3].

In response, metaheuristic algorithms have emerged as powerful alternatives capable of navigating intricate solution landscapes without gradient information [4]. These general-purpose search strategies do not require problem-specific information. Over the past decades, a wide variety of metaheuristics have been proposed, encompassing both metaphor-inspired approaches (e.g., Genetic Algorithms [5], Particle Swarm Optimization [6], Ant Colony Optimization [7]) and non-metaphor-based strategies (e.g., Differential Evolution [8], Simulated Annealing, Tabu Search). The defining feature of a metaheuristic is its generality and problem-independence, not its metaphorical origin [9,10].

Among the many bio-inspired metaheuristics, the Kangaroo Escape Optimizer (KEO) [11] is a relatively recent algorithm that dynamically balances exploration and exploitation by mimicking adaptive escape behaviors of kangaroos such as erratic evasion, long-distance leaps, and deceptive maneuvers. The exploration–exploitation balance is a fundamental challenge in metaheuristic design; excessive exploration can slow convergence, while excessive exploitation may lead to premature convergence to local optima [12,13]. Despite its promising performance, the original KEO, like many population-based optimizers, still faces challenges in maintaining adequate population diversity during the mid-phase search, escaping local optima in highly multimodal environments, and achieving precise convergence in highly constrained nonlinear optimization problems such as pressure vessel design and tubular column design, where the feasible regions are narrow and fragmented [14].

To address these limitations, this paper introduces the Enhanced Kangaroo Escape Optimizer (EKEO), which strategically incorporates two well-established mechanisms: a Differential Evolution (DE) mutation operator [15] and a Quasi-Oppositional Learning (QOL) strategy [16]. DE mutation strengthens local exploitation by directing the search around the current best solution, while QOL enhances global exploration by considering quasi-opposite points of the population to maintain diversity and aid in escape from local traps. These enhancements are embedded into the core KEO framework, creating a synergistic search mechanism that improves both convergence speed and solution quality.

The primary scientific contributions of this work are as follows:

1. A Principled Synergistic Integration of Complementary Search Mechanisms
  We demonstrate the fundamentally complementary nature of a directed exploitation operator via Differential Evolution Mutation (DEM) and a diversity-driven exploration mechanism via Quasi-Oppositional Learning (QOL) in addressing the exploration–exploitation tradeoff. Their integration within the KEO framework is not arbitrary, being grounded in the principle that DEM accelerates convergence around promising solutions while QOL actively maintains population diversity and enables escape from local optima. This synergy yields a robust search dynamic that resolves a key limitation of behavior-based optimizers, which often struggle to balance these competing objectives.
2. Empirical Demonstration of Mechanism Complementarity via Ablation and Causal Analysis
  In addition to reporting benchmark results, we provide systematic empirical evidence of why the integration works. A dedicated ablation study isolates the individual contributions of DEM and QOL, showing that DEM dominates late-stage convergence improvement while QOL primarily contributes to diversity maintenance and local optima escape. This causal analysis transforms the evaluation from a mere performance comparison into a validated demonstration of how complementary mechanisms jointly enhance optimization behavior.
3. A Generalizable Design Principle for Hybrid Metaheuristics
  The integration strategy employed in EKEO couples a best-guided exploitation operator (DE/best/1) with a diversity-preserving exploration operator (quasi-oppositional learning), constituting a design principle that is applicable beyond the specific KEO framework. We articulate this principle and argue that it can be transferred to other population-based optimizers, offering a reusable insight for hybrid algorithm design rather than merely a single algorithm variant.
4. Robustness Validation Under Real-World Constraints as Evidence of Generalizability
  The effectiveness of the proposed synergy is validated on four challenging engineering design problems characterized by nonlinear constraints, mixed discrete–continuous variables, and narrow feasible regions. These problems are selected precisely because they expose the fragility of many algorithmic enhancements that perform well on unconstrained benchmarks but fail under constraints. The consistent superiority of EKEO across these diverse real-world problems provides strong evidence that the synergy between DEM and QOL yields not only benchmark-level performance but also genuine robustness and practical reliability.

The remainder of this paper is organized as follows: Section 2 details the original KEO algorithm and introduces the enhanced EKEO framework, including the rationale behind the integrated strategies and the pseudocode of the proposed algorithm; Section 3 presents the experimental setup, benchmark functions, and comprehensive results on both classical and CEC 2019 test sets, accompanied by statistical and visual analyses; Section 4 demonstrates the practical effectiveness of EKEO on four engineering design problems, with detailed discussions of the obtained results; finally, Section 5 concludes the paper by summarizing the key findings, highlighting the contributions, and outlining potential directions for future research.

## 2. Enhanced Kangaroo Escape Optimizer

KEO is a population-based metaheuristic inspired by the adaptive survival strategies of kangaroos in unpredictable environments [11]. It models the dynamic escape behaviors of kangaroos in order to achieve a balance between global exploration and local exploitation in complex optimization landscapes. The algorithm operates through a structured iterative process, where each candidate solution is represented as a kangaroo and its position in the solution space is updated based on energy-driven behavioral strategies.

### 2.1. Original Kangaroo Escape Optimizer

The KEO algorithm consists of five main components: population initialization, energy dynamics modeling, movement strategies, boundary handling and selection, and iteration control.

#### 2.1.1. Population Initialization

The algorithm begins by randomly initializing a population of *N* agents in a *D*-dimensional bounded search space. The initial position of each agent $P_{i}$ is generated as Equation (1):

(1) $$P_{i}=L+\mathrm{rand}\left(0,1\right)\times \left(U-L\right),\;i=1,2,\ldots ,N$$

where L and U denote the lower and upper bounds of the decision variables, respectively. The fitness of each agent is then evaluated based on the objective function $f(P_{i})$.

#### 2.1.2. Energy Dynamics Model

Each agent is assigned a time-varying energy level E(t) that governs its movement strategy. The energy is modeled using a chaotic logistic map to simulate biological rhythms and environmental stress as Equation (2):

(2) λ(t+1)=4·λ(t)·(1−λ(t)),λ(0)=0.7,

(3) $$E_{i}\left(t\right)=\left[1-\left(\frac{t}{T_{\max}}\cdot \rho_{i,1}\right)\right]\cdot \left(\frac{5\cdot \lambda (t)}{100}+0.95\right),$$

where *t* is the current iteration, $T_{\max}$ is the maximum number of iterations, and $\rho_{i,1}∼U\left(0,1\right)$ is a random number specific to each agent. The energy level starts high to promote extensive exploration and gradually decays to facilitate local refinement.

#### 2.1.3. Movement Strategies

At each iteration, every agent selects one of three strategies based on a random number z∼U(0,1):

- If z<0.5: Safe Zone Search with Decoy Drop (Strategy S)
  The agent moves toward a safe reference point $P_{\mathrm{safe}}$, which is chosen randomly from among three options: (i) random agent (exploration), (ii) local best (exploitation), or (iii) global best $P_{\mathrm{best}}$ (intensive exploitation). The update rule incorporates a decoy mask $M_{\mathrm{decoy}}$ to introduce controlled randomness (Equation (4)).
  (4) $$M_{\mathrm{decoy}}=\left\{\begin{array}{cc} \mathrm{round}\left({\left[U_{1}\left(0,1\right)\right]}_{1\times D}\circ {\left[U_{2}\left(0,1\right)\right]}_{1\times D}\right), & \mathrm{if}\;r_{3}\geq 2/3\\ \mathrm{round}\left({\left[U_{1}\left(0,1\right)\right]}_{1\times D}\right), & \mathrm{if}\;1/3<r_{3}<2/3\\ {\;[1\;1\;\ldots \;1]}_{1\times D}, & \mathrm{otherwise} \end{array}\right.$$
  The structure of the decoy mask is determined by the value of a random variable $r_{3}$ drawn uniformly from the interval [0,1].
  (5) $$P_{i}^{\mathrm{new}}=M_{\mathrm{decoy}}\circ N\left(1,D\right)\circ \left(P_{i}-P_{\mathrm{safe}}\right)+P_{\mathrm{safe}}$$
  The mask $M_{\mathrm{decoy}}$ in Equation (5) is probabilistically generated to partially update dimensions, thereby simulating deceptive tactics and maintaining population diversity.
- If z≥0.5: Energy-Based Escape Strategies
  The agent’s energy $E_{i}\left(t\right)$ is compared to a threshold $E_{\mathrm{thr}}=0.5$:
  - If $E_{i}\left(t\right)\geq 0.5$: Long Jump (Strategy L). The agent performs a large step (Equation (6)) to explore distant regions:
    (6) $$P_{i}^{\mathrm{new}}=2\cdot \left(P_{i}\circ M_{\mathrm{decoy}}\right)\circ N\left(1,D\right)+P_{i}.$$
  - If $E_{i}\left(t\right)<0.5$: Zigzag Motion (Strategy Z). The agent executes erratic localized movements around the current best solution $P_{\mathrm{best}}$. A rotation angle θ is randomly sampled as Equation (7).
    (7) $$\theta =\theta_{\max}\cdot \left(2\rho_{2}-1\right),\;\theta_{\max}=30^{\circ }$$
    (8) $$V_{i}=P_{\mathrm{best}}-P_{i}$$
    (9) $$V_{r}=\sin\left(\theta \right)\cdot U_{⊥}\cdot ∥V_{i}∥+\cos\left(\theta \right)\cdot V_{i}$$
    (10) $$P_{i}^{\mathrm{new}}=P_{i}+\sin\left(\theta \right)\cdot \beta \cdot \left(V_{r}\circ N\left(1,D\right)\right)$$
    Here, $U_{⊥}$ is a unit vector orthogonal to $V_{i}$, β=0.5 controls step size, and N(1,D) is a *D*-dimensional Gaussian random vector.

### 2.2. Proposed EKEO Algorithm

KEO is a population-based metaheuristic that models kangaroo escape behaviors to balance exploration and exploitation; like many population-based optimizers, it can face challenges such as premature convergence in complex multimodal landscapes, loss of diversity during the mid-phase search, and difficulty in balancing convergence speed with solution accuracy, particularly in highly deceptive or constrained problems. To overcome these limitations, this paper proposes an enhanced version called EKEO that strategically incorporates two well-established mechanisms: a differential evolution mutation operator and a quasi-oppositional learning strategy. This integration aims to synergistically improve the algorithm’s global search capability and local refinement power.

#### 2.2.1. Reasons for Enhancement

Despite the competitive performance of the original KEO, its effectiveness in complex multimodal optimization landscapes can be hindered by several factors:

1. Insufficient Local Exploitation: The algorithm’s reliance on biologically inspired movements such as zigzags and long jumps may not provide the intensive directed search needed for precise local refinement near optimal regions in later stages.
2. Premature Convergence: As iterations progress, population diversity can decrease rapidly, leading the search to become trapped in suboptimal basins of attraction. Maintaining adequate diversity during the optimization process is crucial for avoiding premature convergence [17,18].
3. Suboptimal Balance: While innovative, the adaptive switching mechanism based on chaotic energy may not always achieve the most efficient tradeoff between exploring new areas and exploiting known good solutions, especially in high-dimensional or deceptive search spaces [12].

To address these issues, two complementary strategies that target exploitation and exploration are integrated. Enhancement of KEO into EKEO is conceptually aligned with the principle of algorithmic synergy, where complementary search mechanisms are combined to create a more robust optimization framework [19].

#### 2.2.2. Integration of Differential Evolution Mutation

The Differential Evolution Mutation (DEM) strategy [8,15] is known for its strong local search ability and simplicity. It guides the search by leveraging the information of the current best solution and the difference vector between two random individuals, facilitating convergence toward promising regions. Advanced DE variants such as those with parameter adaptation mechanisms have demonstrated strong performance on complex optimization problems [20,21].

During each iteration, the DE/best/1 mutation is activated for each kangaroo (agent) $P_{i}$ with a probability p=0.5. The mutant vector $m_{i}$ is generated as Equation (11):

(11) $$m_{i}=P_{\mathrm{best}}+F\cdot \left(P_{r1}-P_{r2}\right)$$

where $P_{\mathrm{best}}$ is the current global best position, $P_{r1}$ and $P_{r2}$ are two distinct individuals randomly selected from the population $\left(r_{1}\neq r_{2}\neq i\right)$, and F∈[0,1] is a randomly generated scaling factor for the current iteration.

#### 2.2.3. Integration of Quasi-Oppositional Learning

Quasi-Oppositional Learning (QOL) [16,22,23] is a proven technique for enhancing exploration by considering both a solution and its opposite. QOL generates solutions between the center of the search space and the opposite solution, offering a higher probability of finding regions closer to the global optimum. Opposition-based strategies have been successfully integrated into various metaheuristics to improve their exploration capabilities and convergence speed [24].

After the primary position updates of all agents in an iteration, a QOL phase is executed to enhance population diversity and accelerate convergence:

1. For each agent $P_{i}$ in the current population, calculate its opposite point $P_{i}^{\mathrm{opp}}$ as Equation (12):
  (12) $$P_{i}^{\mathrm{opp}}=L+U-P_{i}$$
  where L and U are the lower and upper bounds of the search space, respectively.
2. Compute the quasi-opposite point $P_{i}^{\mathrm{qol}}$ as Equation (13):
  (13) $$P_{i}^{\mathrm{qol}}=\mathrm{rand}\left(\right)\cdot \left(M-P_{i}^{\mathrm{opp}}\right)+P_{i}^{\mathrm{opp}}$$
  where M=(L+U)/2 is the center point of the search space and rand() generates a uniform random number in [0,1].
3. Apply boundary control to ensure that $P_{i}^{\mathrm{qol}}$ remains within [L,U].
4. Perform greedy selection between the original agent $P_{i}$ and its quasi-opposite counterpart $P_{i}^{\mathrm{qol}}$ as Equation (14):
  (14) $$P_{i}^{\mathrm{new}}=\left\{\begin{array}{cc} P_{i}^{\mathrm{qol}}, & \mathrm{if}\;f\left(P_{i}^{\mathrm{qol}}\right)\leq f\left(P_{i}\right)\\ P_{i}, & \mathrm{otherwise} \end{array}\right.$$
  and retain the better candidate for the next generation.

This QOL phase acts as a diversity injection mechanism, helping the algorithm to escape local optima and explore undiscovered regions of the search space more efficiently.

- Enhancing Search Balance: DE mutation focuses on exploitation around $P_{\mathrm{best}}$, while QOL promotes exploration via quasi-opposite points. Their probabilistic activation creates a more robust and adaptive search dynamic.
- Maintaining Efficiency: Both strategies have low computational overhead (O(N·D)), preserving the overall efficiency of the original KEO framework.
- Improving Robustness: The synergistic effect reduces the risk of premature convergence, making EKEO more reliable across different problem landscapes.

#### 2.2.4. Enhanced KEO

The pseudocode illustrating the core workflow of the Enhanced Kangaroo Escape Optimizer (EKEO) is shown in Algorithm 1. The algorithm incorporates an energy-aware mechanism to divide the search process into three distinct strategies: safe-zone search, long-jump escape, and zigzag escape. It integrates several enhancement techniques, including chaotic mapping, a decoy mask mechanism, and differential evolution mutation, while also employing quasi-oppositional learning for boundary handling to balance global exploration and local exploitation. Through iterative optimization, the algorithm outputs the best found solution along with its fitness value, making EKEO suitable for solving continuous optimization problems.
**Algorithm** **1** Pseudocode of the Enhanced Kangaroo Escape Optimizer (EKEO)**Require:**       *N*: Population size (number of kangaroos)      *D*: Dimension of the problem      U,L: Upper and lower bounds of the search space      $T_{\max}$: Maximum number of iterations**Ensure:**       $X_{\mathrm{best}}$: Best found solution      $F_{\mathrm{best}}$: Corresponding fitness value  1.Initialization Phase  2.Initialize population $X_{i}$ by Equation (1)     ▹ Random initialization within bounds  3.Evaluate initial fitness $f(X_{i})$ for i=1,…,N  4.**for** t=1 to $T_{\max}$ **do**  5.    Generate chaotic value λ(t) using logistic map by Equation (2)  6.    **for** i=1 to *N* **do**  7.        Compute energy level $E_{i}\left(t\right)$ by Equation (3)  8.        Generate random number z∼U(0,1)  9.        **if** z<0.5 **then**10.           Strategy S: Safe Zone Search with Decoy Drop      ▹ Exploration-oriented11.           Generate decoy mask $M_{\mathrm{decoy}}$ probabilistically by Equation (4)12.           Update position $X_{i}^{\mathrm{new}}$ by Equation (5)13.        **else**14.           **if** $E_{i}\left(t\right)\geq E_{\mathrm{thr}}$ **then**15.               Strategy L: Long Jump Escape             ▹ Global exploration16.               Generate decoy mask $M_{\mathrm{decoy}}$17.               Update position $X_{i}^{\mathrm{new}}$ by Equation (6)18.           **else**19.               Strategy Z: Zigzag Escape               ▹ Local exploitation20.               Compute $V_{i}=X_{\mathrm{best}}-X_{i}$ by Equation (8)21.               Update position $X_{i}^{\mathrm{new}}$ by Equation (10)22.           **end if**23.           Differential Evolution Mutation           ▹ Enhance local exploitation24.           Generate mutant vector $m_{i}$ by Equation (11)25.           $X_{i}^{\mathrm{new}}\leftarrow m_{i}$ with probability p=0.526.        **end if**27.        Boundary Handling                 ▹ Enforce variable bounds28.        Clip $X_{i}^{\mathrm{new}}$ to [L,U]29.        Quasi-Oppositional Learning            ▹ Enhance population diversity30.        Compute quasi-opposite point $X_{i}^{\mathrm{qol}}$ by Equation (13)31.        Apply greedy selection by Equation (14)32.        Evaluate $f(X_{i}^{\mathrm{new}})$33.        **if** $f\left(X_{i}^{\mathrm{new}}\right)\leq f\left(X_{i}\right)$ **then**34.             $X_{i}\leftarrow X_{i}^{\mathrm{new}}$, $f\left(X_{i}\right)\leftarrow f\left(X_{i}^{\mathrm{new}}\right)$                ▹ Greedy selection35.        **end if**36.    **end for**37.    Update global best $X_{\mathrm{best}}$ and $F_{\mathrm{best}}$38.**end for**39.**return** $X_{\mathrm{best}}$, $F_{\mathrm{best}}$, convergence curve

EKEO addresses key limitations of the original KEO by integrating a Differential Evolution Mutation (DEM) strategy and a Quasi-Oppositional Learning (QOL) mechanism. DEM enhances local exploitation and accelerates convergence, while QOL effectively maintains population diversity and improves global exploration. EKEO provides a powerful tool for solving complex optimization problems. As validated by the experiments reported below, this dual-strategy integration leads to measurable improvements in convergence accuracy, speed, and robustness.

### 2.3. Ablation Experiments

**Figure 1:**
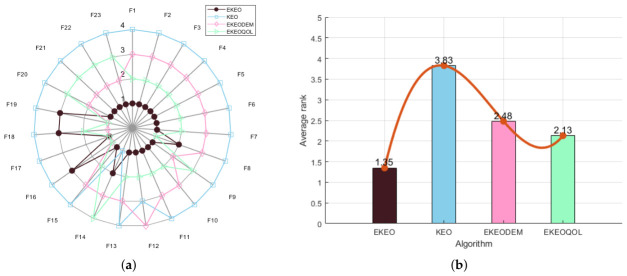
Ablation experiments for 23 classical benchmark functions: (**a**) radar chart and (**b**) rank chart.

**Figure 2:**
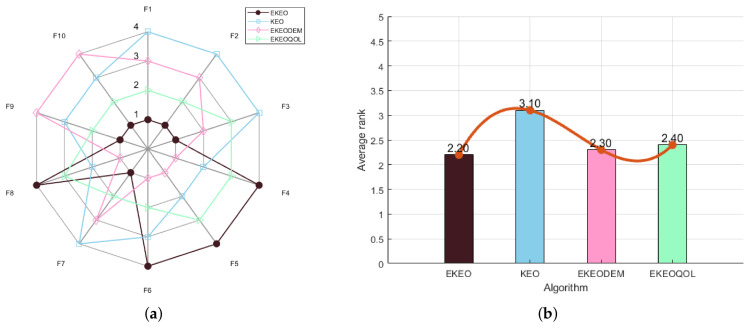
Ablation experiments for CEC 2019: (**a**) radar chart and (**b**) rank chart.

To validate the effectiveness of each enhancement module in the proposed EKEO algorithm, an ablation study is conducted comparing two EKEO variants alongside the original KEO algorithm: EKEO with mutation enhancement only (EKEODEM), and EKEO with opposition-based learning only (EKEQOOL). Performance is evaluated using radar charts (Figure 1a for 23 classical benchmark functions and Figure 2a for CEC 2019), which present normalized scores across multiple benchmark functions, together with and average rank bar plots (Figure 1b and Figure 2b) that summarize the overall ranking of the algorithms.

As illustrated in Figure 1b and Figure 2b, EKEO consistently achieves the lowest average ranks of 1.35 on the 23 classical benchmark functions and 2.20 on the CEC 2019 test suite, substantially outperforming the original KEO (3.83 and 3.10, respectively) and its two variants, EKEODEM (2.48 and 2.30) and EKEQOOL (2.13 and 2.40). The radar charts further demonstrate that EKEO attains near-perfect normalized scores across most benchmark functions, showing high robustness and adaptability. In contrast, KEO exhibits notable performance fluctuations on complex functions. While the variants show certain improvements, they consistently underperform relative to the complete EKEO. These results collectively validate the effectiveness of the proposed enhancement modules.

In summary, EKEO achieves the optimal overall performance across all test functions, with the lowest average rank and the most extensive coverage in the radar charts. This indicates that the two enhancement strategies of mutation enhancement and opposition-based learning work synergistically to improve the algorithm’s performance on both classical functions and the CEC 2019 test suite. These results confirm the critical contributions of each module in enhancing the algorithm’s search capability and convergence accuracy.

### 2.4. Time Complexity Analysis

The time complexity of the proposed EKEO algorithm is analyzed as follows. Let *N* be the population size, *D* the problem dimension, $T_{\max}$ the maximum number of iterations, and O(f) the cost of a single fitness evaluation.

During initialization, generating *N* random agents in *D* dimensions costs O(N·D) plus O(N·O(f)) for initial fitness evaluation. In each iteration, the algorithm performs the following for every agent: energy updating (O(1)), one of the three movement strategies (O(D)), boundary clipping (O(D)), and a differential evolution mutation (O(D)) with probability 0.5. Then, the quasi-oppositional learning phase computes an opposite and quasi-opposite point (O(D) each), followed by a greedy selection that requires one additional fitness evaluation (O(f)). Finally, updating the global best takes O(N) per iteration.

Summing over all iterations, the total asymptotic complexity is

$$O\left(\mathrm{EKEO}\right)=O\left(T_{\max}\cdot N\cdot D\right)\;+\;O\left(2\cdot T_{\max}\cdot N\cdot O\left(f\right)\right).$$

Ignoring constants, this simplifies to $O\left(T_{\max}\cdot N\cdot \left(D+O\left(f\right)\right)\right)$. Compared to the original KEO, which uses one fitness evaluation per agent per iteration, EKEO requires approximately twice as many fitness evaluations due to the incorporation of QOL greedy selection. However, as shown in Section 3 and Section 4, the gains in convergence speed and solution quality typically reduce the required number of iterations, offsetting this modest overhead.

## 3. Experimental Results and Discussion

To evaluate the performance of EKEO, a multi-stage experimental framework is constructed. The evaluation begins with preliminary validation using standard benchmark functions, followed by extensive assessment on 23 classical benchmark functions (unimodal (F1, F3), basic multimodal (F6, F7, F10), hybrid (F12, F15), and composite (F17, F19, F20, F21, F23)) along with the CEC 2019 benchmark suite [25]. This dual-test-platform strategy effectively validates the algorithm’s performance across heterogeneous problem scenarios. All comparative algorithms are executed in a strictly controlled computational environment with identical hardware configurations and parameter settings to ensure fairness and reproducibility.

### 3.1. Experimental Setup

Selecting appropriate comparison algorithms is crucial for obtaining scientifically valid and comparable results. In order to thoroughly assess the performance of the proposed EKEO and identify potential improvement areas, eleven mature and novel metaheuristic algorithms are selected for the comparative study. These include the standard Kangaroo Escape Optimizer (KEO) [11], the Artificial Lemming Algorithm (ALA) [26], the Water Uptake and Transport in Plants algorithm (WUTP) [27], the Fata Morgana Algorithm (FATA) [28], Particle Swarm Optimization (PSO) [6], the GOOSE algorithm (GOOSE) [29], the Genetic Algorithm (GA) [5], the Grey Wolf Optimizer (GWO) [30], the Whale Optimization Algorithm (WOA) [31], the Dung Beetle Optimizer (DBO) [32], and the Artificial Protozoa Optimizer (APO) [33].

The experiments were conducted using 23 classical benchmark functions and the CEC 2019 test suite. All algorithms were implemented in MATLAB 2024a and run on a workstation equipped with an Intel^®^ Core™ i7-13700K processor and 32 GB RAM running Windows 11. Experimental parameters were selected through systematic analysis to achieve a balance between computational efficiency and optimization performance. The population size was set to 30, offering a compromise between computational cost and population diversity. The maximum number of iterations was set to 500, providing sufficient convergence time for the algorithms to fully manifest performance differences. Each experimental condition was independently repeated 30 times, ensuring statistical reliability and providing sufficient power for subsequent non-parametric hypothesis testing.

### 3.2. Results and Analysis on the 23 Classical Test Functions

The 23 benchmark functions are categorized into four problem types: unimodal (F1), basic multimodal (F2–F8), hybrid (F9–F13), and composite (F14–F23). As shown in Table A1 and Table A2, EKEO demonstrates superior overall performance across all categories in Table 1 and Table 2. On functions such as F1, F3, F9, F10,and F11, EKEO achieves the global optimum (zero or near-zero values), outperforming all other algorithms. For instance, On F5, a multimodal function, EKEO attains the lowest minimum value ($2.54\times 10^{1}$) and smallest standard deviation ($4.36\times 10^{-1}$), indicating its high accuracy and consistency.

Regarding stability, EKEO consistently exhibits the lowest or near-lowest standard deviation across numerous functions. On F6, EKEO’s standard deviation is only $5.96\times 10^{-6}$, significantly lower than that of KEO ($1.01\times 10^{-2}$), ALA ($5.70\times 10^{-2}$), and PSO ($1.76\times 10^{1}$). Similarly, EKEO records the smallest std value on F7 ($2.11\times 10^{-6}$), underscoring its stability in noisy or complex landscapes.

For complex multimodal and composite functions (e.g., F8, F21–F23), EKEO maintains highly competitive results. On F8, although FATA achieves the best average value ($-1.26\times 10^{4}$), EKEO still delivers a competitive average of $-7.73\times 10^{3}$ with moderate stability. On the high-dimensional multimodal functions F21–F23, EKEO achieves the best average fitness values ($-9.90\times 10^{0}$, $-1.02\times 10^{1}$, and $-9.42\times 10^{0}$, respectively) and lowest standard deviations, demonstrating the ability to escape local optima and navigate complex search spaces.

Compared to other algorithms, EKEO consistently outperforms its predecessor KEO across all metrics, validating the effectiveness of enhancement via DEM and QOL. EKEO shows substantial improvements in both accuracy and stability compared to classical algorithms such as PSO, GA, and GWO. On F13, EKEO achieves an average of $2.77\times 10^{0}$ with a std of $7.43\times 10^{-1}$, while GA yields an average of $9.62\times 10^{5}$ with extreme variability, highlighting EKEO’s efficiency on irregular and rugged landscapes.

Although a few algorithms such as FATA and DBO occasionally achieve competitive results on specific functions (e.g., F8 and F9), EKEO maintains a leading position across a broader range of benchmarks, especially in terms of comprehensive performance.

In summary, EKEO shows strong solution accuracy, robustness, and consistent stability across diverse function types, including unimodal, multimodal, and fixed-dimensional composite functions. Its performance validates the algorithm’s well-balanced tradeoff between global exploration and local exploitation, establishing it as a high-performance general-purpose optimizer for complex continuous optimization problems.

Table 2 presents the Wilcoxon rank-sum test results (significance level α=0.05) comparing EKEO with 12 other algorithms across 23 benchmark functions. The analysis demonstrates that EKEO exhibits statistically significant superiority on the vast majority of test problems [34,35].

EKEO shows significant performance advantages (p<0.05) over all or nearly all competitors on 18 out of 23 functions (78.3%). On key functions such as F1–F4, F6–F7, and F9–F12, the *p*-values reach the extreme lower bound of detection (e.g., $1.21\times 10^{-12}$), confirming that EKEO’s superiority is not only numerical but also statistically robust. Compared to its predecessor KEO, EKEO significantly outperforms on 18 functions, with particularly strong evidence on F1–F13. This validates the effectiveness of EKEO’s enhanced mechanisms. Against classical algorithms such as PSO, GA, GWO, and WOA, EKEO maintains dominance on 19–21 functions (82.6–91.3%). On complex multimodal functions (F13, F18–F23), EKEO retains strong statistical advantages, especially against GA on F21–F23 ($p<10^{-11}$). Although no significant difference is observed on a few functions (such as F14, F16, F17, F20, and F23) against specific algorithms, these exceptions do not diminish EKEO’s overall leading position.

In summary, the Wilcoxon test provides rigorous statistical evidence that EKEO’s performance improvements are significant, consistent, and particularly pronounced on complex optimization problems, supporting its advancement as a high-performance metaheuristic optimizer.

These statistical findings complement the numerical results in Table 1 and Table 3, confirming that EKEO’s performance advantages are not only quantitatively substantial but also statistically robust and reliable across diverse problem types. The extremely low *p*-values observed across most function comparisons provide strong evidence that EKEO represents a statistically significant advancement in metaheuristic optimization methodology.

**Figure 3:**
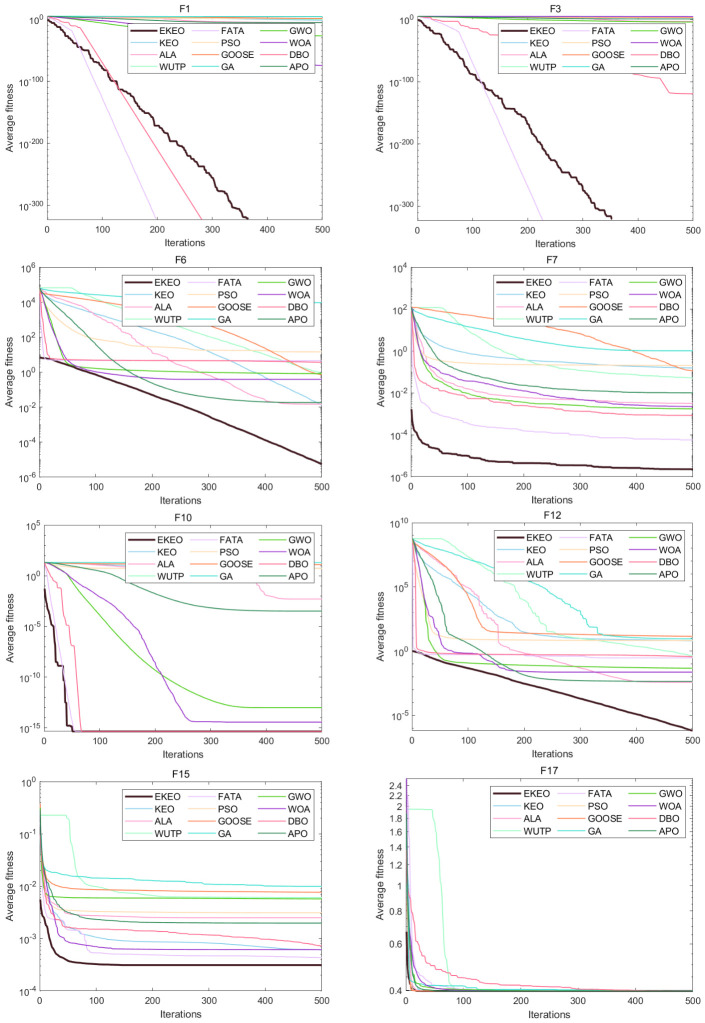
Convergence analysis on a subset of the 23 benchmark functions.

**Figure 4:**
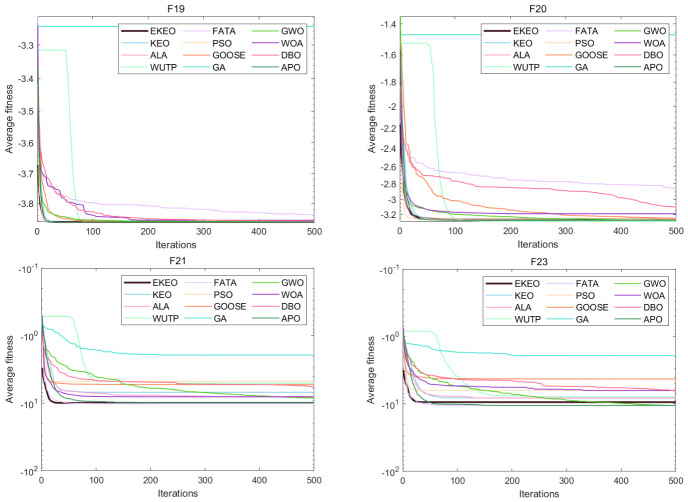
Convergence analysis on a subset of the 23 benchmark functions.

Based on a comprehensive analysis of 12 convergence curves (a subset of the 23), as shown in Figure 3 and Figure 4, this study reveals the performance characteristics and differences among various algorithms from the perspective of the dynamic optimization process. EKEO demonstrates strong overall convergence performance. On unimodal and structurally clear multimodal problems (e.g., F1, F3, F7, F10), the algorithm exhibits rapid convergence, approaching the theoretical optimum in the early iterations. On complex multimodal and composite functions (e.g., F6, F15, F21, F23), its convergence trajectory remains smooth and stable, consistently maintaining a leading downward trend. This reflects the ability of EKEO to avoid local optima and maintain robustness. The convergence analysis further validates the effectiveness of the improvement strategies; chaotic mapping enhances the guidance of global exploration in the early stages, while differential evolution and quasi-oppositional learning ensure the depth and stability of local exploitation in the later stages.

In terms of algorithm comparison, EKEO shows first-tier superiority in convergence speed, accuracy, and stability. Algorithms such as FATA and DBO show competitiveness on certain problems; however, their convergence curves often exhibit noticeable fluctuations or stagnation in later stages, indicating insufficient stability. In contrast, traditional algorithms such as PSO and GA show premature convergence or low search efficiency on most complex problems. Their convergence trajectories often become flat or stagnant, highlighting the advancements of search mechanism design in newer metaheuristics.

The dynamic characteristics of the convergence curves further reveal the intrinsic search behaviors of different algorithms. EKEO maintains efficient search and continuous improvement across all stages without showing obvious performance tradeoffs. In comparison, other algorithms often struggle to balance exploration and exploitation; some are overly aggressive, leading to entrapment in local optima, while others are overly conservative, resulting in slow convergence. These observations corroborate the statistical conclusions from the tabular data, collectively indicating that EKEO not only achieves superior solutions but also unifies search efficiency, convergence accuracy, and stability in the optimization process. In this way, it provides a reliable and efficient solution for complex continuous optimization problems.

**Figure 5:**
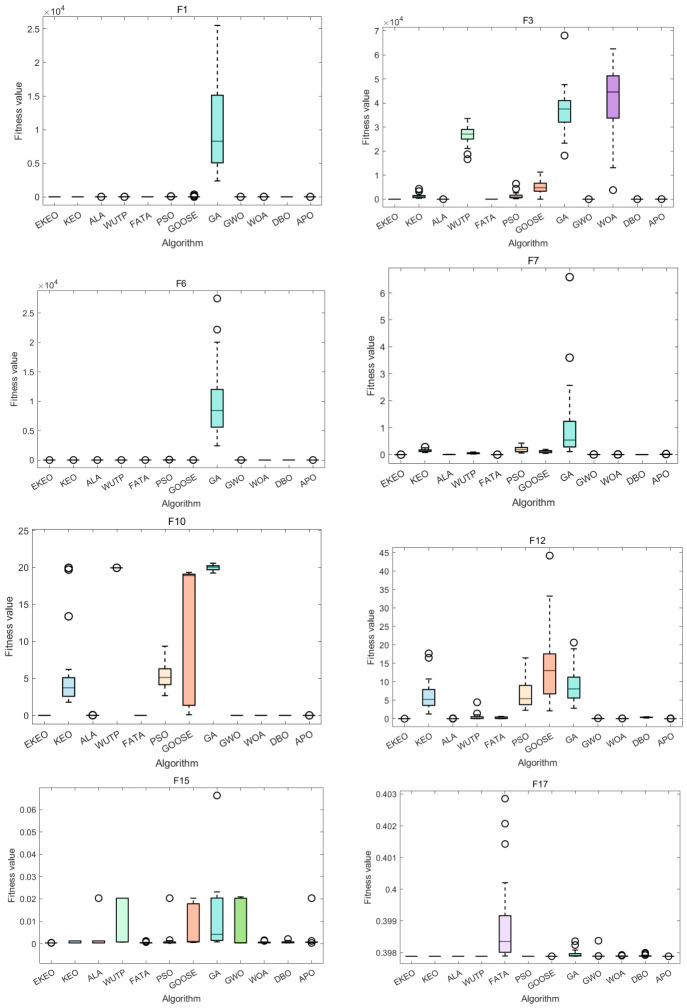
ANOVA test results on a subset of the 23 benchmark functions.

**Figure 6:**
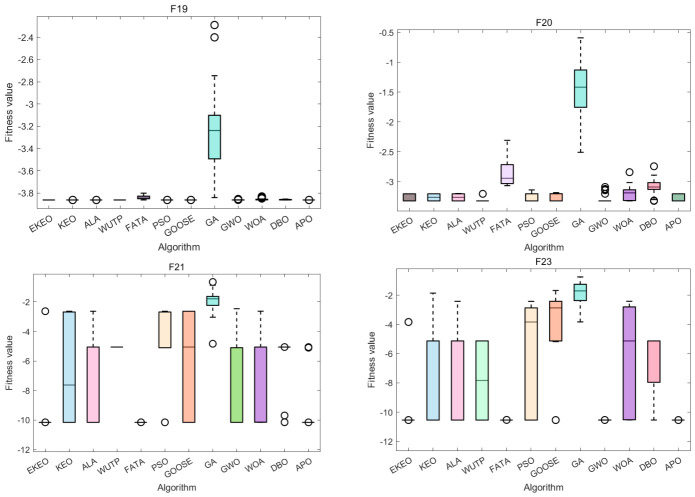
ANOVA test results on a subset of the 23 benchmark functions.

An ANOVA-based performance distribution analysis using box plots highlights algorithm-specific characteristics, as shown in Figure 5 and Figure 6. A clear performance hierarchy emerges among the evaluated algorithms. EKEO consistently exhibits the lowest median fitness values and the narrowest Inter-Quartile Ranges (IQRs) across all test functions. This indicates not only good solution quality, but also high stability and robustness by minimizing performance variation across independent runs. In contrast, other algorithms display varying degrees of dispersion and median elevation; traditional methods such as PSO and GA show particularly wide distributions and frequent outliers, especially on complex functions such as F21 and F23.

The influence of function complexity on algorithm performance is clearly visualized through these boxplots. Most algorithms demonstrate relatively compact distributions on simpler unimodal functions, though EKEO remains notably more concentrated. As problem complexity increases, particularly in high-dimensional, multimodal, and composite functions, the performance spread widens significantly for all algorithms except EKEO, which maintains a consistently narrow IQR. This highlights EKEO’s enhanced ability to avoid local optima and sustain stable convergence behavior even in challenging search landscapes.

Overall, the boxplot analysis provides strong visual confirmation of EKEO’s dual strengths in solution accuracy and algorithmic reliability. Its consistently tight performance distributions reflect low outcome variability, a critical feature for practical applications requiring dependable optimization results. These findings align with and reinforce the earlier numerical and statistical evaluations, collectively underscoring EKEO’s position as a robust and high-performance optimizer suitable for a wide range of complex continuous optimization problems.

**Figure 7:**
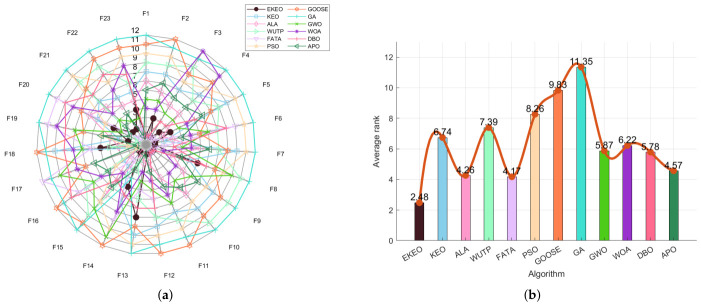
Performance ranking of optimization algorithms on the 23 benchmark functions: (**a**) radar chart and (**b**) average rank chart.

Based on the integrated analysis of the ranking chart and radar diagram in Figure 7, EKEO shows comprehensive superiority across the benchmark suite. EKEO achieves the lowest average rank in the ranking chart (4.17), significantly outperforming its predecessor KEO (8.26) and all traditional algorithms. The radar diagram further reveals that EKEO’s performance polygon is consistently closest to the center across all 23 functions, indicating balanced and robust performance regardless of problem type. This visual evidence aligns with and reinforces previous quantitative results, confirming EKEO as a well-rounded and highly competitive optimizer for diverse complex continuous optimization tasks.

The Friedman omnibus test is a non-parametric statistical test used to detect significant differences across multiple algorithms over multiple benchmark functions, serving as a global test before performing post hoc pairwise comparisons:

$$\chi_{F}^{2}=\frac{12N}{k(k+1)}\left(\sum_{j=1}^{k}{\bar{R}}_{j}^{2}-\frac{k{\left(k+1\right)}^{2}}{4}\right),$$

where:

- *N* is the total number of benchmark functions.
- *k* is the number of algorithms being compared.
- ${\bar{R}}_{j}$ is the average rank of the *j*-th algorithm across all functions, as shown in Figure 7b.

The test statistic follows a chi-square distribution with df=k−1 degrees of freedom under the null hypothesis:

$$\chi_{F}^{2}\approx 98.81,\;df=11.$$

The Friedman test statistic is $\chi_{F}^{2}=98.81$ with df=11, which is far greater than the critical value of the chi-square distribution at any common significance level (e.g., at α=0.001, the critical value is approximately 31.26). This implies that the corresponding *p*-value is far below 0.001 (p≪0.001). Therefore, the null hypothesis is rejected at the α=0.05 significance level. This confirms that there is a statistically significant global difference among the 12 algorithms on the 23 benchmark functions.

Based on a comprehensive experimental evaluation across the 23 benchmark functions, EKEO achieves significantly better solution accuracy with high consistency, convergence stability, and statistical significance. The algorithm achieves results that are close to the theoretical optimum on multiple function types, while its exceptionally low standard deviations and compact boxplot distributions confirm its strong robustness. The Wilcoxon rank-sum test further validates its performance advantage from a statistical perspective, with *p*-values below $10^{-5}$ in the majority of comparisons. Convergence curves indicate that EKEO combines rapid convergence with sustained refinement capability. Moreover, its top average ranking score (4.17) and well-balanced performance profile across the radar chart collectively illustrate consistent performance and algorithmic equilibrium across diverse problem categories.

### 3.3. Results and Analysis for CEC 2019

Table 3 summarizes the performance of algorithms on the CEC 2019 benchmark. EKEO converges precisely and stably to the theoretical optimum (1) on functions F1, F4–F8, and F10, while other algorithms yield results ranging from $10^{0}$ to $10^{6}$, a gap of 3–6 orders of magnitude. For example, on F1, KEO, PSO, and GA achieve optima of $6.67\times 10^{3}$, $5.09\times 10^{3}$, and $3.82\times 10^{6}$, respectively. EKEO also exhibits near-zero standard deviations across most functions, indicating consistent convergence over 30 independent runs, which reduces practical uncertainty. In contrast, the other algorithms show high variability: GOOSE has a standard deviation of $1.30\times 10^{9}$ on F1, and even advanced algorithms such as FATA and DBO have significantly higher variances than EKEO (on F2, FATA achieves std = $4.99\times 10^{-2}$ vs. EKEO’s $1.26\times 10^{-1}$). Most competitors exhibit an imbalance consisting of acceptable minima but poor averages and large stds (e.g., GA on F1: min = $3.82\times 10^{6}$, avg = $3.50\times 10^{7}$, std = $2.59\times 10^{7}$), indicating frequent local optima trapping. Compared to KEO, EKEO achieves order-of-magnitude improvements on functions such as F1, F7, and F10, confirming that DEM and QOL form a synergistic framework that enhances global search and convergence stability.

**Figure 8:**
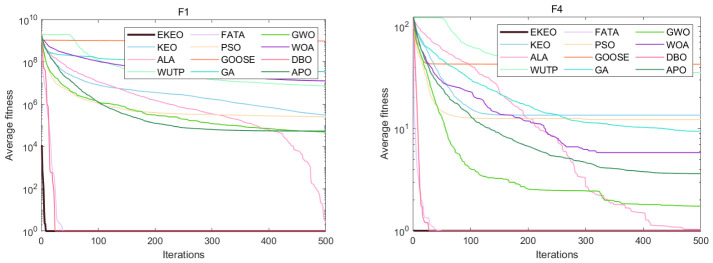

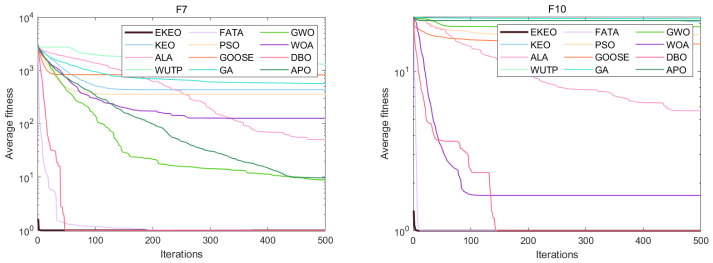
Convergence analysis on a subset of the CEC 2019 functions.

**Figure 9:**
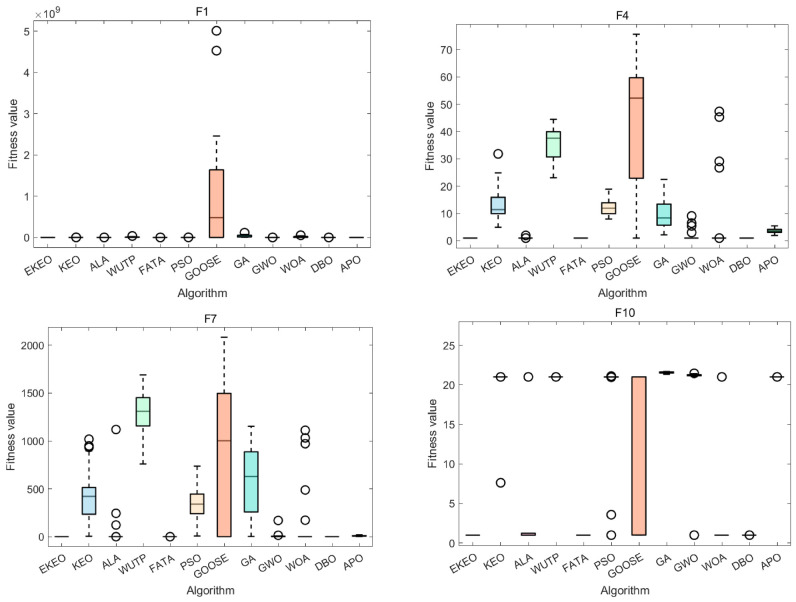
ANOVA test results on a subset of the CEC 2019 functions.

The Wilcoxon rank-sum test results (Table 4) demonstrate that EKEO achieves statistically significant performance superiority on the CEC 2019 benchmark set. The *p*-values are consistently below $10^{-9}$ when compared with most competing algorithms (including KEO, PSO, and GA), reaching as low as $10^{-12}$ on several functions. On functions with a broad global optimum region (e.g., F4–F6), no significant difference is observed between EKEO and advanced algorithms such as FATA and DBO (p≈1), which is expected when multiple algorithms stably converge to the optimum. However, EKEO shows clear statistical superiority on more complex functions (e.g., F1, F2, F7, F8). These extremely low *p*-values allow for rejection of the null hypothesis of equivalent performance. Convergence curves (Figure 8) show that EKEO converges quickly and stably on most functions, avoids local optima, and maintains a leading position throughout iterations, outperforming traditional algorithms such as PSO and GA. Reproducibility and stability are further supported by low standard deviations (Table 3) and compact boxplots (Figure 9), while practical relevance is demonstrated on four engineering problems (Section 4).

From the boxplots in Figure 9, it is evident that EKEO shows the most concentrated and optimal-biased performance distribution across multiple functions (e.g., F1, F10). Its boxes are compact with low medians and very few outliers, reflecting high consistency and stability across multiple independent runs. In comparison, the boxes of other algorithms are generally longer and more dispersed, particularly for traditional algorithms such as GA, PSO, and GOOSE, which display numerous high outliers on several functions. This indicates high result variability and low reliability. Although advanced algorithms such as FATA and DBO achieve medians close to EKEO on certain functions such as F8 and F10, their boxes are wider. This indicates noticeable performance fluctuations, suggesting that these algorithms are less capable than EKEO in stably reproducing high-quality solutions.

**Figure 10:**
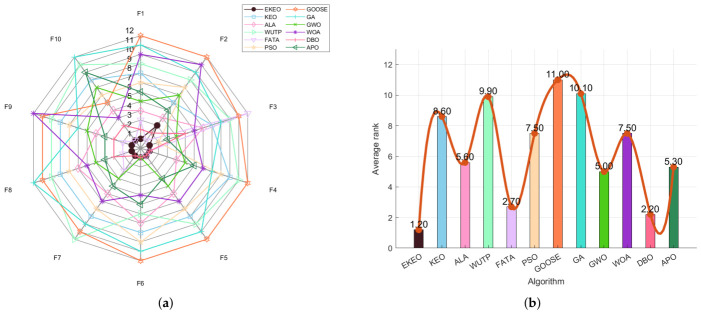
Performance ranking on the CEC 2019 benchmark: (**a**) radar chart and (**b**) average rank chart.

Convergence curves and boxplots show that EKEO converges faster and more steadily than competitors, with strong robustness across repeated runs. Advanced algorithms such as FATA and DBO compete on specific problems but lack EKEO’s overall stability and cross-function consistency. Traditional algorithms such as PSO and GA lag in convergence speed, accuracy, and stability. As shown in Figure 10, EKEO achieves the best average rank (2.20), far outperforming the second-ranked DBO (5.30). Its radar polygon is consistently closest to the center, indicating balanced performance across all functions, while others show gaps on complex problems such as F1 and F7. In summary, EKEO demonstrates leading convergence accuracy, robustness, and consistent performance, validating its competitiveness as an advanced optimizer.

EKEO integrates differential evolution mutation and quasi-oppositional learning into a synergistic framework, achieving a strong balance between global exploration and local exploitation. On the 23 classical benchmark functions, it stably converges to the theoretical or near-optimum solutions on most problems and achieves standard deviations near zero, indicating high accuracy and stability. Boxplot analysis confirms concentrated performance distributions and few outliers, reflecting robustness and repeatability. On the more challenging CEC 2019 suite, EKEO reaches or approaches the global optimum across various function types, with fast and steady convergence. Wilcoxon rank-sum tests confirm statistical significance and the ranking analysis along with radar plots consistently places EKEO first overall, demonstrating balanced performance across all problem categories without obvious weaknesses.

### 3.4. Comparison with Recent Hybrid Algorithms

In summary, this study validates that EKEO achieves unified improvements in solution quality, convergence stability, and algorithmic reliability through systematic numerical comparisons, statistical tests, and multi-dimensional visual analysis. Its performance advantage is reflected not only in the numerical results but also in the predictability of the search process and reproducibility of the outcomes, marking a key transition from ‘potentially better’ to ‘reliably better’. Therefore, EKEO can be regarded as an efficient, robust, and advanced metaheuristic solver suitable for complex continuous optimization problems, with reliable potential for solving real-world engineering optimization tasks.

## 4. Engineering Application

To evaluate the engineering application performance of the improved algorithm, a systematic assessment of a set of optimization algorithms is conducted across four representative engineering design problems. The tested algorithms include EKEO, KEO, ALA, WUTP, FATA, PSO, GOOSE, GA, GWO, WOA, DBO, and APO. Building on the enhanced mechanisms of EKEO, which integrates DEM and QOL into a cohesive framework, this experiment aims to verify whether such synergistic strategies can effectively balance global exploration and local exploitation under real-world constraints [1,14]. Through rigorous comparative analysis on step-cone pulley design, gear system design, tubular column design, and pressure vessel design problems, this work assesses not only final design quality but also convergence behavior, constraint satisfaction, and algorithmic robustness, thereby providing insights into the applicability and competitiveness of modern metaheuristics in practical engineering contexts [36,37].

### 4.1. Step-Cone Pulley Design

**Figure 11:**
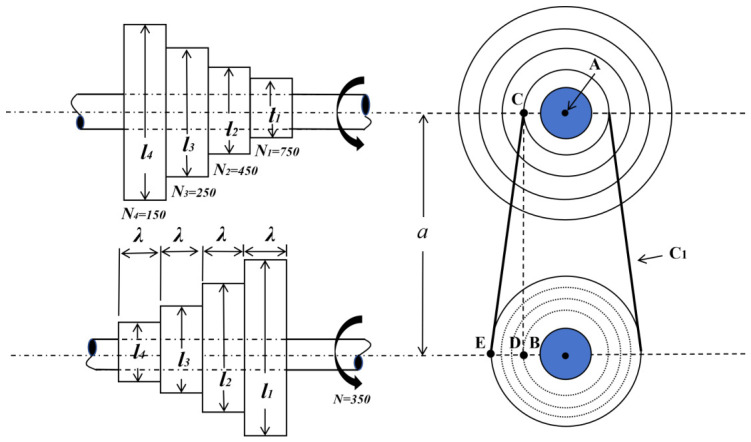
Step-cone pulley design mechanical structure diagram.

The step-cone pulley design is a classic constrained mechanical optimization problem that aims to minimize the weight of a four-stage stepped pulley system. As illustrated in Figure 11, the system configuration typically involves multiple pulley stages with varying diameters $\left(l_{1},l_{2},l_{3},l_{4}\right)$ and a common pulley width ω. The optimization problem is formulated to minimize the total mass of the pulley assembly while satisfying 11 nonlinear constraints that ensure the transmission of a required power output of 0.75 horsepower across each stage [38].

The constraints include both equality and inequality types. The equality constraints $h_{1}$ to $h_{3}$ enforce geometric consistency across pulley stages, while the inequality constraints $g_{5}$ to $g_{8}$ ensure that the transmitted power at each stage meets or exceeds the required 0.75 HP (0.75×745.6998 W). The power transmission for each stage $P_{i}$ depends on belt tension, friction coefficient μ, belt dimensions, and rotational parameters, incorporating nonlinear terms such as the wrap angle and friction-based slip factor $R_{i}$.

Given the presence of multiple nonlinear interactions among design variables, especially between diameters, belt lengths, and power equations, this problem poses significant challenges in maintaining feasibility while converging to a globally optimal design. As such, it serves as a rigorous benchmark for evaluating the constraint-handling capability, convergence stability, and solution precision of modern metaheuristic optimizers such as EKEO and its counterparts.

Minimize

(15) $$\begin{array}{c} f\left(\bar{x}\right)=\rho \omega \left(\frac{l_{1}^{2}}{1+{\left(\frac{N_{1}}{N}\right)}^{2}}+\frac{l_{2}^{2}}{1+{\left(\frac{N_{2}}{N}\right)}^{2}}+\frac{l_{3}^{2}}{1+{\left(\frac{N_{3}}{N}\right)}^{2}}+\frac{l_{4}^{2}}{1+{\left(\frac{N_{4}}{N}\right)}^{2}}\right), \end{array}$$

where $l_{i}$ denotes the length associated with each stage, $N_{i}$ the rotational speeds, and ρ the material density.

The above is subject to

(16) $$\begin{array}{c} h_{1}\left(\bar{x}\right)=C_{1}-C_{2}=0,\\ h_{2}\left(\bar{x}\right)=C_{1}-C_{3}=0,\\ h_{3}\left(\bar{x}\right)=C_{1}-C_{4}=0,\\ g_{5}\left(\bar{x}\right)=0.75\times 745.6998-P_{1}\leq 0,\\ g_{6}\left(\bar{x}\right)=0.75\times 745.6998-P_{2}\leq 0,\\ g_{7}\left(\bar{x}\right)=0.75\times 745.6998-P_{3}\leq 0,\\ g_{8}\left(\bar{x}\right)=0.75\times 745.6998-P_{4}\leq 0, \end{array}$$

where

(17) $$\begin{array}{c} C_{i}=\pi l_{i}^{2}\left[1+\frac{N_{i}}{N}+\frac{{\left(\frac{N_{i}}{N}-1\right)}^{2}}{4a}+\frac{2}{a}\right],\;i=1,2,3,4,\\ R_{i}=\exp\left[\mu \left(\pi -2\sin^{-1}\left(\frac{\frac{N_{i}}{N}-1}{\frac{l_{i}}{2a}}\right)\right)\right],\;i=1,2,3,4,\\ P_{i}=\frac{st\omega \left(1-R_{i}\right)\pi l_{i}N_{i}}{60},\;i=1,2,3,4 \end{array}$$

with parameters

(18) $$\begin{array}{c} t=8\;\mathrm{mm},\;s=1.75\;\mathrm{MPa},\;\mu =0.35,\;\rho =7200\;{\mathrm{kg}/m}^{3},\;a=3\;\mathrm{mm}. \end{array}$$

**Figure 12:**
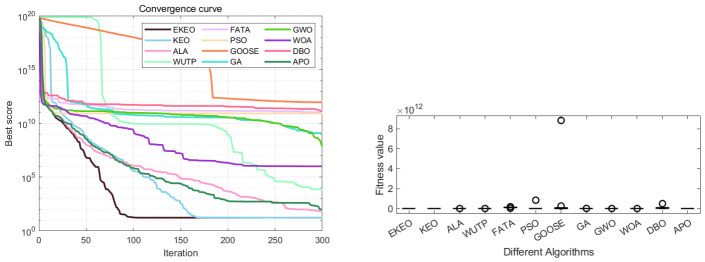
Convergence curve and ANOVA test graph of step-cone pulley design optimization results.

EKEO shows comprehensively superior performance on the step-cone pulley design optimization problem as shown in Table 5 and Table 6, Figure 12. Its convergence curve indicates that the algorithm can rapidly and stably approach the global optimum, achieving the lowest objective function value (16.21) while satisfying all complex constraints with feasible design parameters. Statistical results show that EKEO’s standard deviation (0.64) is significantly lower than those of other algorithms, indicating highly stable and reliable outcomes. Boxplot analysis further confirms its concentrated performance distribution with no outliers. In contrast, traditional algorithms (e.g., PSO, GA) and some modern algorithms (e.g., FATA, DBO) exhibit convergence oscillations, large result fluctuations, and even deterioration by orders of magnitude, highlighting the effectiveness and robustness of EKEO in solving multivariable and strongly constrained nonlinear engineering optimization problems.

### 4.2. Gear Train Design

**Figure 13:**
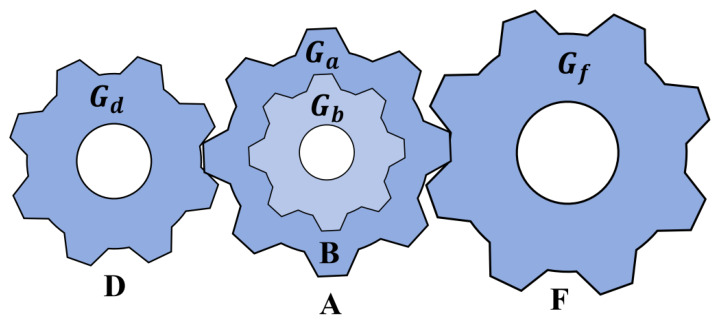
Gear train design mechanical structure diagram.

The gear train design problem is a classic discrete unconstrained optimization problem in which the objective is to minimize the deviation between the actual transmission ratio and a target value by optimizing the number of teeth of four gears ($G_{a}$, $G_{b}$, $G_{d}$, $G_{f}$), thereby reducing the design and manufacturing costs of the transmission system. As shown in Figure 13, the gear train consists of four gears, and its transmission ratio is determined by $\frac{G_{b}\cdot G_{d}}{G_{a}\cdot G_{f}}$ [39]. The optimization objective can be formulated as minimizing the squared error between the actual transmission ratio and the theoretical value 1/6.931.

Consider

(19) $$\bar{x}=\left[G_{a},G_{b},G_{d},G_{f}\right]$$

and minimize

(20) $$\begin{array}{c} \min f\left(\bar{x}\right)={\left(\frac{1}{6.931}-\frac{G_{b}\cdot G_{d}}{G_{a}\cdot G_{f}}\right)}^{2} \end{array}$$

subject to

(21) $$s.t.=\left\{\begin{array}{cc} x_{i}\in N_{+}, & i=1,2,3,4,\\ 0.01\leq x_{i}\leq 60, & i=1,2,3,4. \end{array}\right.$$

**Figure 14:**
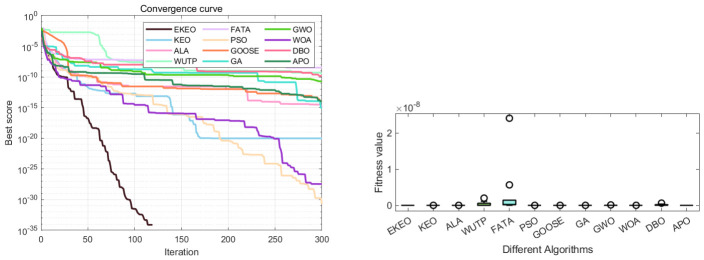
Convergence curve and ANOVA test graph of gear train design optimization results.

In the discrete integer optimization problem involving gear train design, EKEO demonstrates strong comprehensive performance as shown in Table 7 and Table 8. It converges to the theoretical optimum (objective function value of 0) at a fast rate while maintaining zero-error results across all 30 independent runs, with its standard deviation of zero reflecting high stability and repeatability. Convergence curves and boxplots further confirm its efficient search process and completely concentrated result distribution in Figure 14. In contrast, although some algorithms can achieve near-optimal solutions, they fall short of EKEO in terms of stability, convergence speed, and result consistency, with algorithms such as FATA and GOOSE exhibiting noticeable fluctuations and outliers. These results validate that EKEO’s chaotic initialization and quasi-oppositional learning mechanisms effectively coordinate global exploration and local refinement when addressing discrete nonlinear optimization problems, highlighting its suitability for engineering applications.

### 4.3. Tubular Column Design

**Figure 15:**
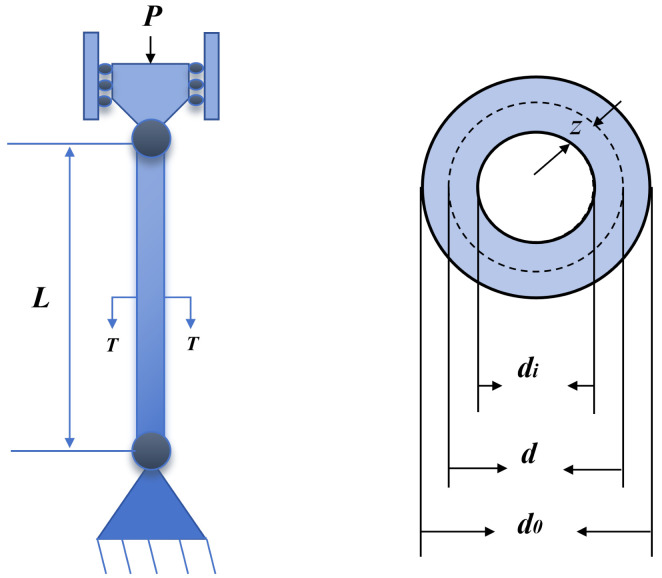
Tubular column design mechanical structure diagram.

The tubular column design problem is a classic engineering structural optimization problem that aims to determine the cross-sectional dimensions of a cylindrical column to minimize manufacturing cost while satisfying mechanical performance and geometric constraints. As illustrated in Figure 15, the column is a thin-walled tubular structure with two design variables: mean diameter $x_{1}$, denoted as *d*, and wall thickness $x_{2}$, denoted as *z* [37]. The objective function is to minimize the material and manufacturing costs, expressed as shown below.

Consider

(22) $$\bar{X}={\left[x_{1},x_{2}\right]}^{⊤}$$

and minimize

(23) $$\begin{array}{c} f\left(\bar{X}\right)=9.82x_{1}x_{2}+2x_{1} \end{array}$$

subject to

(24) $$\begin{array}{c} g_{1}\left(\bar{X}\right)=\frac{P}{\pi x_{1}x_{2}\sigma_{y}}-1\leq 0,\\ g_{2}\left(\bar{X}\right)=\frac{8PL^{2}}{\pi^{3}Ex_{1}x_{2}\left(x_{1}^{2}+x_{2}^{2}\right)}-1\leq 0,\\ g_{3}\left(\bar{X}\right)=\frac{2.0}{x_{1}}-1\leq 0,\\ g_{4}\left(\bar{X}\right)=\frac{x_{1}}{14}-1\leq 0,\\ g_{5}\left(\bar{X}\right)=\frac{0.2}{x_{2}}-1\leq 0,\\ g_{6}\left(\bar{X}\right)=\frac{x_{2}}{0.9}-1\leq 0 \end{array}$$

with bounds

(25) $$\begin{array}{c} 2\leq x_{1}\leq 14,\;0.2\leq x_{2}\leq 0.8, \end{array}$$

where

(26) $$P=\mathrm{applied}\;\mathrm{load},\;L=\mathrm{length},\;\sigma_{y}=\mathrm{yield}\;\mathrm{stress},\;E=\mathrm{Young}’s\;\mathrm{modulus}.$$

**Figure 16:**
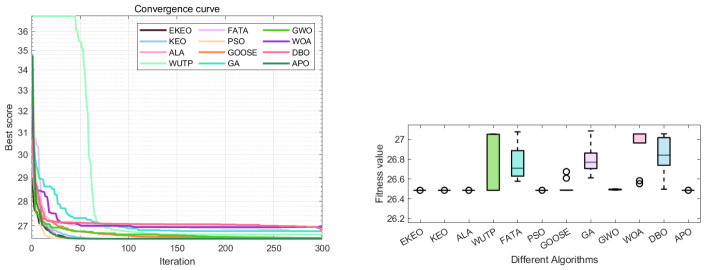
Convergence curve and ANOVA test graph of tubular column design optimization results.

Based on the optimization parameters and statistical indicator results, EKEO shows the best performance in the tubular column design application in Table 9 and Table 10. Among all algorithms, EKEO, KEO, ALA, PSO, GOOSE, GWO, and APO achieve the same minimum objective function value $f(\bar{x})=26.49$; however, EKEO ranks first and exhibits a standard deviation of 0.00 with identical mean, median, and minimum values, indicating high stability and consistency. In contrast, FATA, GA, WOA, and DBO perform slightly worse in terms of optimal value, worst value, standard deviation, and mean, in particular showing poorer stability and consistency. Overall, EKEO surpasses the others in convergence (Figure 16), with its better robustness and reliability in practical engineering applications making it suitable for structural design scenarios that require high accuracy and system stability.

### 4.4. Pressure Vessel Design

**Figure 17:**
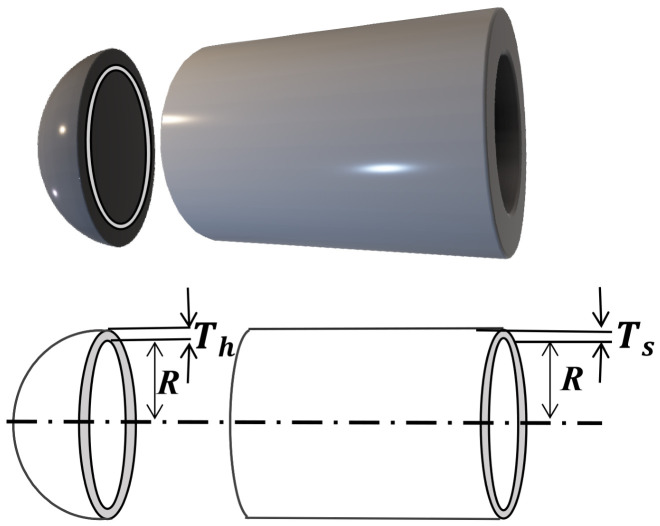
Mechanical structure diagram of the pressure vessel design problem.

In optimal design of pressure vessels, the objective is to minimize the total manufacturing cost by formulating a constrained optimization problem involving four design variables (Figure 17). The design variables are the head thickness $T_{s}$ (denoted as $x_{1}$), the shell thickness $T_{h}$ (denoted as $x_{2}$), the inner radius *R* (denoted as $x_{3}$), and the cylindrical section length *L* (denoted as $x_{4}$). Among these, $x_{1}$ and $x_{2}$ are discrete variables, taking integer multiples of 0.0625, while $x_{3}$ and $x_{4}$ are continuous variables [40].

Minimize

(27) $$\begin{array}{c} f\left(\bar{x}\right)=1.7781x_{2}x_{3}^{2}+0.6224x_{1}x_{3}x_{4}+3.1661x_{1}^{2}x_{4}+19.84x_{1}^{2}x_{3} \end{array}$$

subject to

(28) $$\begin{array}{c} g_{1}\left(\bar{x}\right)=0.00954x_{3}\leq x_{2},\\ g_{2}\left(\bar{x}\right)=0.0193x_{3}\leq x_{1},\\ g_{3}\left(\bar{x}\right)=x_{4}\leq 240,\\ g_{4}\left(\bar{x}\right)=-\pi x_{3}^{2}x_{4}-\frac{4}{3}\pi x_{3}^{3}\leq -1296000 \end{array}$$

with bounds

(29) $$\begin{array}{c} 10\leq x_{4},x_{3}\leq 200,\\ 1\leq x_{2},x_{1}\leq 99\;(\mathrm{integer}\;\mathrm{variables}). \end{array}\;$$

Based on the optimization parameters and statistical results of the pressure vessel design problem as shown in Table 11 and Table 12, EKEO shows the best overall performance in solving this mixed-variable nonlinear constrained optimization problem. Among all the tested algorithms, EKEO, KEO, ALA, WUTP, PSO, and GWO achieve the same minimum objective function value $f\left(\bar{x}\right)=6.06\times 10^{3}$, indicating comparable ability to locate the optimal solution. However, EKEO ranks first in terms of ranking, stability, and consistency. In addition, it exhibits the lowest standard deviation ($2.21\times 10^{2}$) among all algorithms together with the best mean ($6.24\times 10^{3}$) and median ($6.09\times 10^{3}$) values. This confirms that EKEO not only converges to the global optimum but also shows strong robustness and repeatability.

In contrast, algorithms such as FATA, GOOSE, GA, WOA, and DBO perform poorly in terms of optimal value, worst value, standard deviation, and mean. Notably, GOOSE yields an optimal value of $1.32\times 10^{4}$, which is significantly higher than others, and its standard deviation reaches $9.12\times 10^{3}$, indicating unstable convergence behavior and a tendency to become trapped in local optima or exhibit high variability.

**Figure 18:**
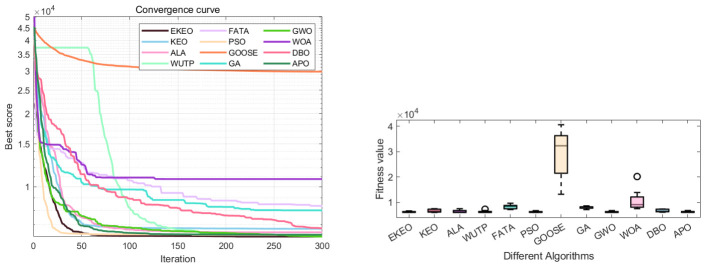
Convergence curve and ANOVA test graph of pressure vessel design optimization results.

The convergence curve further illustrates that EKEO converges rapidly during iteration and attains a final value substantially lower than those of FATA, GOOSE, and other algorithms in Figure 18. In summary, for the pressure vessel design—a typical engineering optimization problem—EKEO outperforms the others in convergence accuracy, stability, and computational robustness, making it suitable for real-world engineering applications that require high reliability in design.

In our systematic evaluation of four representative engineering optimization problems, EKEO consistently shows strong overall performance. In step-cone pulley design, gear train design, tubular column design, and pressure vessel design, EKEO not only converges stably to the theoretical optimum or near-optimal solutions but also significantly outperforms the compared algorithms in terms of convergence speed, stability, and robustness. Its standard deviation is generally the lowest and the result distribution is highly concentrated, indicating good repeatability and engineering applicability. For complex engineering problems involving mixed variables, strong constraints, and nonlinearity, EKEO effectively balances global exploration and local exploitation by integrating quasi-oppositional learning and differential evolution mutation mechanisms, thereby avoiding premature convergence and result fluctuations. Consequently, EKEO can be regarded as an efficient, reliable, and practically applicable metaheuristic optimization method for engineering design, offering significant value in scenarios requiring high precision and high stability.

## 5. Conclusions and Prospects

In addition to the demonstrated performance gains, this work contributes three scientific insights. First, it provides empirical evidence that directed exploitation (DEM) and diversity-preserving exploration (QOL) are fundamentally complementary, with their synergistic integration resolving the exploration–exploitation tradeoff more effectively than either mechanism alone. Second, it offers a causal explanation for this synergy through ablation analysis, showing the distinct phase-specific contributions of each component. Third, it articulates a generalizable design principle by coupling a best-guided exploitation operator with a diversity-preserving exploration operator, which can inform hybrid algorithm design beyond the specific EKEO framework. These contributions shift the focus from metaphor-based enhancement to mechanism-driven design, aligning with the field’s evolution toward principled algorithmic development. Experimental results indicate that the developed EKEO exhibits better convergence accuracy, faster convergence speed, and stronger stability across 23 classical test functions, the CEC 2019 benchmark suite, and four representative engineering optimization problems.

To further enhance the applicability of EKEO and expand its scope, future research could focus on the following directions:

1. Multi-Objective Extension: Develop a multi-objective variant of EKEO based on Pareto dominance and diversity preservation mechanisms to address engineering optimization problems with conflicting objectives.
2. Dynamic Environment Adaptation: Incorporate time-varying parameters and online learning strategies that would enable the algorithm to adapt to dynamic optimization problems where objective functions or constraints change over time.
3. Hybrid Intelligent Optimization: Integrate EKEO with machine learning surrogate models to construct a surrogate-assisted hybrid optimization framework, which would improve solution efficiency on computationally expensive problems.
4. Comparative Studies with Advanced Hybrid Algorithms: Comprehensive comparisons between EKEO and recently proposed high-performance hybrid algorithms (e.g., LSHADE with CMA-ES [21], DG2 [19], and other state-of-the-art DE variants) on both benchmark functions and real-world engineering problems.

As an advanced metaheuristic algorithm that integrates complementary search mechanisms, EKEO demonstrates good balance and adaptability in complex constrained engineering optimization, providing a reliable and efficient optimization tool for practical engineering applications. Subsequent research could further extend its application dimensions and enhance computational efficiency while maintaining the exploration–exploitation balance, which could better address tasks involving complex system optimization and intelligent decision-making.

## Figures and Tables

**Table 1:** Performance comparison of optimization algorithms on 23 benchmark functions.

Func.	Res.	EKEO	KEO	ALA	WUTP	FATA	PSO	GOOSE	GA	GWO	WOA	DBO	APO
F1	min	$0.00\times 10^{0}$	$4.46\times 10^{-3}$	$7.81\times 10^{-7}$	$1.80\times 10^{-1}$	$0.00\times 10^{0}$	$7.20\times 10^{-1}$	$3.16\times 10^{-3}$	$2.37\times 10^{3}$	$4.22\times 10^{-29}$	$4.11\times 10^{-82}$	$0.00\times 10^{0}$	$4.85\times 10^{-7}$
	std	$0.00\times 10^{0}$	$1.10\times 10^{-2}$	$4.80\times 10^{-6}$	$5.40\times 10^{-1}$	$0.00\times 10^{0}$	$1.66\times 10^{1}$	$6.66\times 10^{1}$	$6.90\times 10^{3}$	$2.48\times 10^{-27}$	$8.67\times 10^{-75}$	$0.00\times 10^{0}$	$1.74\times 10^{-6}$
	avg	$0.00\times 10^{0}$	$1.68\times 10^{-2}$	$5.43\times 10^{-6}$	$8.37\times 10^{-1}$	$0.00\times 10^{0}$	$1.13\times 10^{1}$	$1.91\times 10^{1}$	$1.04\times 10^{4}$	$1.55\times 10^{-27}$	$2.59\times 10^{-75}$	$0.00\times 10^{0}$	$2.58\times 10^{-6}$
F2	min	$0.00\times 10^{0}$	$4.23\times 10^{-3}$	$3.25\times 10^{-4}$	$1.84\times 10^{-1}$	$0.00\times 10^{0}$	$6.58\times 10^{-1}$	$4.70\times 10^{-1}$	$2.25\times 10^{1}$	$1.37\times 10^{-17}$	$3.79\times 10^{-61}$	$0.00\times 10^{0}$	$5.38\times 10^{-4}$
	std	$0.00\times 10^{0}$	$2.34\times 10^{-2}$	$4.26\times 10^{-4}$	$2.49\times 10^{-1}$	$0.00\times 10^{0}$	$2.90\times 10^{0}$	$9.17\times 10^{2}$	$8.67\times 10^{0}$	$9.84\times 10^{-17}$	$9.55\times 10^{-50}$	$0.00\times 10^{0}$	$4.16\times 10^{-4}$
	avg	$0.00\times 10^{0}$	$2.20\times 10^{-2}$	$8.86\times 10^{-4}$	$6.61\times 10^{-1}$	$0.00\times 10^{0}$	$3.80\times 10^{0}$	$2.54\times 10^{2}$	$3.90\times 10^{1}$	$1.13\times 10^{-16}$	$2.81\times 10^{-50}$	$0.00\times 10^{0}$	$1.17\times 10^{-3}$
F3	min	$0.00\times 10^{0}$	$3.56\times 10^{2}$	$7.47\times 10^{-5}$	$1.66\times 10^{4}$	$0.00\times 10^{0}$	$1.81\times 10^{2}$	$2.06\times 10^{0}$	$1.81\times 10^{4}$	$5.72\times 10^{-9}$	$3.77\times 10^{3}$	$0.00\times 10^{0}$	$8.89\times 10^{-1}$
	std	$0.00\times 10^{0}$	$8.70\times 10^{2}$	$8.06\times 10^{-4}$	$3.99\times 10^{3}$	$0.00\times 10^{0}$	$1.29\times 10^{3}$	$2.64\times 10^{3}$	$8.75\times 10^{3}$	$3.53\times 10^{-4}$	$1.41\times 10^{4}$	$3.00\times 10^{-119}$	$4.87\times 10^{0}$
	avg	$0.00\times 10^{0}$	$1.28\times 10^{3}$	$9.58\times 10^{-4}$	$2.70\times 10^{4}$	$0.00\times 10^{0}$	$1.38\times 10^{3}$	$5.03\times 10^{3}$	$3.72\times 10^{4}$	$7.83\times 10^{-5}$	$4.18\times 10^{4}$	$5.48\times 10^{-120}$	$7.38\times 10^{0}$
F4	min	$0.00\times 10^{0}$	$8.74\times 10^{0}$	$4.26\times 10^{-3}$	$5.70\times 10^{0}$	$0.00\times 10^{0}$	$7.45\times 10^{0}$	$1.17\times 10^{-1}$	$5.56\times 10^{1}$	$5.96\times 10^{-8}$	$5.15\times 10^{0}$	$0.00\times 10^{0}$	$2.23\times 10^{-2}$
	std	$0.00\times 10^{0}$	$3.36\times 10^{0}$	$5.70\times 10^{-3}$	$3.79\times 10^{0}$	$0.00\times 10^{0}$	$5.03\times 10^{0}$	$2.27\times 10^{1}$	$8.05\times 10^{0}$	$1.49\times 10^{-6}$	$2.80\times 10^{1}$	$1.42\times 10^{-71}$	$2.40\times 10^{-2}$
	avg	$0.00\times 10^{0}$	$1.43\times 10^{1}$	$1.31\times 10^{-2}$	$1.03\times 10^{1}$	$0.00\times 10^{0}$	$1.63\times 10^{1}$	$2.60\times 10^{1}$	$7.19\times 10^{1}$	$9.00\times 10^{-7}$	$5.20\times 10^{1}$	$2.59\times 10^{-72}$	$5.56\times 10^{-2}$
F5	min	$2.54\times 10^{1}$	$8.28\times 10^{1}$	$2.47\times 10^{1}$	$6.48\times 10^{1}$	$1.80\times 10^{-1}$	$1.82\times 10^{2}$	$2.51\times 10^{1}$	$4.34\times 10^{4}$	$2.60\times 10^{1}$	$2.71\times 10^{1}$	$2.81\times 10^{1}$	$2.74\times 10^{1}$
	std	$4.36\times 10^{-1}$	$2.14\times 10^{2}$	$4.19\times 10^{-1}$	$1.17\times 10^{2}$	$8.42\times 10^{0}$	$1.65\times 10^{4}$	$4.35\times 10^{2}$	$2.34\times 10^{6}$	$8.44\times 10^{-1}$	$5.00\times 10^{-1}$	$2.61\times 10^{-1}$	$2.60\times 10^{1}$
	avg	$2.63\times 10^{1}$	$2.34\times 10^{2}$	$2.55\times 10^{1}$	$2.30\times 10^{2}$	$2.53\times 10^{1}$	$3.79\times 10^{3}$	$2.05\times 10^{2}$	$1.21\times 10^{6}$	$2.71\times 10^{1}$	$2.80\times 10^{1}$	$2.87\times 10^{1}$	$3.48\times 10^{1}$
F6	min	$6.18\times 10^{-7}$	$2.04\times 10^{-3}$	$9.67\times 10^{-6}$	$9.67\times 10^{-2}$	$3.03\times 10^{-1}$	$3.19\times 10^{-1}$	$6.11\times 10^{-3}$	$2.41\times 10^{3}$	$2.51\times 10^{-1}$	$6.58\times 10^{-2}$	$2.99\times 10^{0}$	$8.61\times 10^{-4}$
	std	$5.96\times 10^{-6}$	$1.01\times 10^{-2}$	$5.70\times 10^{-2}$	$6.01\times 10^{-1}$	$1.37\times 10^{0}$	$1.76\times 10^{1}$	$3.32\times 10^{0}$	$5.78\times 10^{3}$	$3.33\times 10^{-1}$	$1.77\times 10^{-1}$	$3.76\times 10^{-1}$	$4.13\times 10^{-2}$
	avg	$5.48\times 10^{-6}$	$1.34\times 10^{-2}$	$1.51\times 10^{-2}$	$8.67\times 10^{-1}$	$4.57\times 10^{0}$	$1.46\times 10^{1}$	$7.39\times 10^{-1}$	$9.63\times 10^{3}$	$8.35\times 10^{-1}$	$3.94\times 10^{-1}$	$3.72\times 10^{0}$	$1.84\times 10^{-2}$
F7	min	$6.39\times 10^{-8}$	$7.95\times 10^{-2}$	$8.98\times 10^{-4}$	$2.13\times 10^{-2}$	$7.61\times 10^{-7}$	$5.86\times 10^{-2}$	$4.74\times 10^{-2}$	$1.13\times 10^{-1}$	$4.13\times 10^{-4}$	$1.05\times 10^{-4}$	$5.26\times 10^{-5}$	$3.48\times 10^{-3}$
	std	$2.11\times 10^{-6}$	$5.32\times 10^{-2}$	$1.59\times 10^{-3}$	$1.95\times 10^{-2}$	$5.45\times 10^{-5}$	$1.03\times 10^{-1}$	$4.37\times 10^{-2}$	$1.32\times 10^{0}$	$1.13\times 10^{-3}$	$2.47\times 10^{-3}$	$6.97\times 10^{-4}$	$3.93\times 10^{-3}$
	avg	$2.27\times 10^{-6}$	$1.56\times 10^{1}$	$3.13\times 10^{-3}$	$5.07\times 10^{-2}$	$5.70\times 10^{-5}$	$2.04\times 10^{1}$	$1.14\times 10^{-1}$	$1.03\times 10^{0}$	$1.72\times 10^{-3}$	$2.25\times 10^{-3}$	$8.37\times 10^{-4}$	$1.01\times 10^{-2}$
F8	min	$-9.57\times 10^{3}$	$-9.44\times 10^{3}$	$-1.18\times 10^{4}$	$-5.86\times 10^{3}$	$-1.26\times 10^{4}$	$-8.23\times 10^{3}$	$-8.57\times 10^{3}$	$-3.31\times 10^{3}$	$-7.85\times 10^{3}$	$-1.26\times 10^{4}$	$-1.21\times 10^{4}$	$-9.91\times 10^{3}$
	std	$6.70\times 10^{2}$	$4.96\times 10^{2}$	$1.22\times 10^{3}$	$4.10\times 10^{2}$	$3.23\times 10^{-2}$	$8.09\times 10^{2}$	$7.22\times 10^{2}$	$4.81\times 10^{2}$	$6.19\times 10^{2}$	$1.82\times 10^{3}$	$1.28\times 10^{3}$	$4.65\times 10^{2}$
	avg	$-7.73\times 10^{3}$	$-8.59\times 10^{3}$	$-9.38\times 10^{3}$	$-5.16\times 10^{3}$	$-1.26\times 10^{4}$	$-6.57\times 10^{3}$	$-7.14\times 10^{3}$	$-2.19\times 10^{3}$	$-6.15\times 10^{3}$	$-1.05\times 10^{4}$	$-7.34\times 10^{3}$	$-8.97\times 10^{3}$
F9	min	$0.00\times 10^{0}$	$3.48\times 10^{1}$	$1.47\times 10^{-5}$	$1.85\times 10^{2}$	$0.00\times 10^{0}$	$3.12\times 10^{1}$	$7.77\times 10^{1}$	$2.30\times 10^{2}$	$5.68\times 10^{-14}$	$0.00\times 10^{0}$	$0.00\times 10^{0}$	$2.55\times 10^{1}$
	std	$0.00\times 10^{0}$	$2.24\times 10^{1}$	$1.00\times 10^{0}$	$1.14\times 10^{1}$	$0.00\times 10^{0}$	$1.87\times 10^{1}$	$4.84\times 10^{1}$	$3.20\times 10^{1}$	$5.26\times 10^{0}$	$1.04\times 10^{-14}$	$0.00\times 10^{0}$	$6.72\times 10^{0}$
	avg	$0.00\times 10^{0}$	$6.42\times 10^{1}$	$4.65\times 10^{-1}$	$2.15\times 10^{2}$	$0.00\times 10^{0}$	$6.03\times 10^{1}$	$1.61\times 10^{2}$	$2.75\times 10^{2}$	$3.64\times 10^{0}$	$1.90\times 10^{-15}$	$0.00\times 10^{0}$	$3.59\times 10^{1}$
F10	min	$4.44\times 10^{-16}$	$1.78\times 10^{0}$	$5.62\times 10^{-4}$	$2.00\times 10^{1}$	$4.44\times 10^{-16}$	$2.68\times 10^{0}$	$8.15\times 10^{-2}$	$1.93\times 10^{1}$	$7.51\times 10^{-14}$	$4.44\times 10^{-16}$	$4.44\times 10^{-16}$	$1.48\times 10^{-4}$
	std	$0.00\times 10^{0}$	$5.31\times 10^{0}$	$6.80\times 10^{-3}$	$8.90\times 10^{-4}$	$0.00\times 10^{0}$	$1.61\times 10^{0}$	$8.72\times 10^{0}$	$3.75\times 10^{-1}$	$1.36\times 10^{-14}$	$2.53\times 10^{-15}$	$0.00\times 10^{0}$	$1.16\times 10^{-4}$
	avg	$4.44\times 10^{-16}$	$5.54\times 10^{0}$	$5.05\times 10^{-3}$	$2.00\times 10^{1}$	$4.44\times 10^{-16}$	$5.42\times 10^{0}$	$1.26\times 10^{1}$	$2.00\times 10^{1}$	$9.79\times 10^{-14}$	$3.64\times 10^{-15}$	$4.44\times 10^{-16}$	$3.25\times 10^{-4}$
F11	min	$0.00\times 10^{0}$	$2.74\times 10^{-3}$	$1.27\times 10^{-6}$	$3.62\times 10^{-1}$	$0.00\times 10^{0}$	$4.38\times 10^{-1}$	$8.36\times 10^{-4}$	$2.85\times 10^{1}$	$0.00\times 10^{0}$	$0.00\times 10^{0}$	$0.00\times 10^{0}$	$5.75\times 10^{-7}$
	std	$0.00\times 10^{0}$	$2.93\times 10^{-2}$	$1.41\times 10^{-5}$	$1.77\times 10^{-1}$	$0.00\times 10^{0}$	$2.20\times 10^{-1}$	$2.17\times 10^{2}$	$4.88\times 10^{1}$	$2.07\times 10^{-2}$	$3.42\times 10^{-2}$	$0.00\times 10^{0}$	$3.06\times 10^{-3}$
	avg	$0.00\times 10^{0}$	$5.21\times 10^{-2}$	$1.42\times 10^{-5}$	$7.84\times 10^{-1}$	$0.00\times 10^{0}$	$1.09\times 10^{0}$	$2.25\times 10^{2}$	$9.97\times 10^{1}$	$8.25\times 10^{-3}$	$6.24\times 10^{-3}$	$0.00\times 10^{0}$	$6.34\times 10^{-4}$
F12	min	$9.93\times 10^{-8}$	$1.28\times 10^{0}$	$4.28\times 10^{-6}$	$4.09\times 10^{-3}$	$2.67\times 10^{-3}$	$2.29\times 10^{0}$	$2.13\times 10^{0}$	$2.84\times 10^{0}$	$1.59\times 10^{-2}$	$6.95\times 10^{-3}$	$1.89\times 10^{-1}$	$2.28\times 10^{-5}$
	std	$5.73\times 10^{-7}$	$3.87\times 10^{0}$	$1.90\times 10^{-2}$	$8.38\times 10^{-1}$	$2.14\times 10^{-1}$	$3.84\times 10^{0}$	$9.38\times 10^{0}$	$4.30\times 10^{0}$	$2.27\times 10^{-2}$	$1.69\times 10^{-2}$	$7.84\times 10^{-2}$	$1.89\times 10^{-2}$
	avg	$6.34\times 10^{-7}$	$6.27\times 10^{0}$	$3.70\times 10^{-3}$	$4.40\times 10^{-1}$	$2.95\times 10^{-1}$	$6.66\times 10^{0}$	$1.41\times 10^{1}$	$9.00\times 10^{0}$	$4.66\times 10^{-2}$	$2.31\times 10^{-2}$	$3.66\times 10^{-1}$	$4.36\times 10^{-3}$
F13	min	$2.14\times 10^{-2}$	$7.74\times 10^{-1}$	$2.35\times 10^{-4}$	$6.61\times 10^{-2}$	$1.80\times 10^{-2}$	$9.35\times 10^{0}$	$2.02\times 10^{-3}$	$2.44\times 10^{1}$	$1.06\times 10^{-1}$	$1.11\times 10^{-1}$	$2.44\times 10^{0}$	$5.52\times 10^{-4}$
	std	$7.43\times 10^{-1}$	$1.01\times 10^{1}$	$2.18\times 10^{-1}$	$2.28\times 10^{0}$	$9.69\times 10^{-1}$	$1.18\times 10^{1}$	$1.16\times 10^{1}$	$2.35\times 10^{6}$	$2.42\times 10^{-1}$	$2.51\times 10^{-1}$	$8.22\times 10^{-2}$	$1.32\times 10^{-2}$
	avg	$2.77\times 10^{0}$	$2.43\times 10^{1}$	$1.25\times 10^{-1}$	$1.96\times 10^{0}$	$1.44\times 10^{0}$	$2.96\times 10^{1}$	$4.46\times 10^{0}$	$9.62\times 10^{5}$	$6.71\times 10^{-1}$	$5.23\times 10^{-1}$	$2.68\times 10^{0}$	$1.22\times 10^{-2}$
F14	min	$9.98\times 10^{-1}$	$9.98\times 10^{-1}$	$9.98\times 10^{-1}$	$9.98\times 10^{-1}$	$9.98\times 10^{-1}$	$9.98\times 10^{-1}$	$9.98\times 10^{-1}$	$9.98\times 10^{-1}$	$9.98\times 10^{-1}$	$9.98\times 10^{-1}$	$9.98\times 10^{-1}$	$9.98\times 10^{-1}$
	std	$8.77\times 10^{-1}$	$2.30\times 10^{0}$	$8.25\times 10^{-17}$	$9.98\times 10^{-1}$	$9.98\times 10^{-1}$	$9.98\times 10^{-1}$	$5.93\times 10^{0}$	$3.78\times 10^{-10}$	$4.41\times 10^{0}$	$3.23\times 10^{0}$	$7.61\times 10^{-1}$	$1.11\times 10^{-15}$
	avg	$1.89\times 10^{0}$	$2.48\times 10^{0}$	$9.98\times 10^{-1}$	$8.77\times 10^{-1}$	$2.30\times 10^{0}$	$8.25\times 10^{-17}$	$1.24\times 10^{1}$	$9.98\times 10^{-1}$	$4.91\times 10^{0}$	$3.16\times 10^{0}$	$2.07\times 10^{0}$	$9.98\times 10^{-1}$
F15	min	$3.08\times 10^{-4}$	$3.08\times 10^{-4}$	$3.08\times 10^{-4}$	$6.85\times 10^{-4}$	$3.10\times 10^{-4}$	$3.08\times 10^{-4}$	$5.11\times 10^{-4}$	$7.84\times 10^{-4}$	$3.08\times 10^{-4}$	$3.08\times 10^{-4}$	$3.08\times 10^{-4}$	$3.13\times 10^{-4}$
	std	$2.13\times 10^{-5}$	$4.15\times 10^{-4}$	$6.07\times 10^{-3}$	$8.78\times 10^{-3}$	$1.94\times 10^{-4}$	$6.89\times 10^{-3}$	$8.86\times 10^{-3}$	$1.35\times 10^{-2}$	$9.02\times 10^{-3}$	$3.48\times 10^{-4}$	$4.20\times 10^{-4}$	$5.00\times 10^{-3}$
	avg	$3.11\times 10^{-4}$	$6.09\times 10^{-4}$	$2.50\times 10^{-3}$	$6.06\times 10^{-3}$	$4.34\times 10^{-4}$	$3.10\times 10^{-3}$	$7.68\times 10^{-3}$	$9.96\times 10^{-3}$	$5.74\times 10^{-3}$	$6.15\times 10^{-4}$	$7.23\times 10^{-4}$	$1.98\times 10^{-3}$
F16	min	$-1.03\times 10^{0}$	$-1.03\times 10^{0}$	$-1.03\times 10^{0}$	$-1.03\times 10^{0}$	$-1.03\times 10^{0}$	$-1.03\times 10^{0}$	$-1.03\times 10^{0}$	$-1.03\times 10^{0}$	$-1.03\times 10^{0}$	$-1.03\times 10^{0}$	$-1.03\times 10^{0}$	$-1.03\times 10^{0}$
	std	$5.83\times 10^{-16}$	$6.25\times 10^{-16}$	$6.18\times 10^{-16}$	$2.16\times 10^{-6}$	$1.36\times 10^{-6}$	$6.71\times 10^{-16}$	$2.82\times 10^{-1}$	$1.34\times 10^{-1}$	$2.34\times 10^{-8}$	$6.10\times 10^{-10}$	$4.38\times 10^{-5}$	$3.25\times 10^{-15}$
	avg	$-1.03\times 10^{0}$	$-1.03\times 10^{0}$	$-1.03\times 10^{0}$	$-1.03\times 10^{0}$	$-1.03\times 10^{0}$	$-1.03\times 10^{0}$	$-9.23\times 10^{-1}$	$-9.56\times 10^{-1}$	$-1.03\times 10^{0}$	$-1.03\times 10^{0}$	$-1.03\times 10^{0}$	$-1.03\times 10^{0}$
F17	min	$3.98\times 10^{-1}$	$3.98\times 10^{-1}$	$3.98\times 10^{-1}$	$3.98\times 10^{-1}$	$3.98\times 10^{-1}$	$3.98\times 10^{-1}$	$3.98\times 10^{-1}$	$3.98\times 10^{-1}$	$3.98\times 10^{-1}$	$3.98\times 10^{-1}$	$3.98\times 10^{-1}$	$3.98\times 10^{-1}$
	std	$0.00\times 10^{0}$	$0.00\times 10^{0}$	$0.00\times 10^{0}$	$0.00\times 10^{0}$	$1.28\times 10^{-3}$	$0.00\times 10^{0}$	$9.05\times 10^{-10}$	$1.18\times 10^{-4}$	$8.89\times 10^{-5}$	$1.27\times 10^{-5}$	$2.36\times 10^{-5}$	$5.14\times 10^{-10}$
	avg	$3.98\times 10^{-1}$	$3.98\times 10^{-1}$	$3.98\times 10^{-1}$	$3.98\times 10^{-1}$	$3.99\times 10^{-1}$	$3.98\times 10^{-1}$	$3.98\times 10^{-1}$	$3.98\times 10^{-1}$	$3.98\times 10^{-1}$	$3.98\times 10^{-1}$	$3.98\times 10^{-1}$	$3.98\times 10^{-1}$
F18	min	$3.00\times 10^{0}$	$3.00\times 10^{0}$	$3.00\times 10^{0}$	$3.00\times 10^{0}$	$3.00\times 10^{0}$	$3.00\times 10^{0}$	$3.00\times 10^{0}$	$3.00\times 10^{0}$	$3.00\times 10^{0}$	$3.00\times 10^{0}$	$3.00\times 10^{0}$	$3.00\times 10^{0}$
	std	$4.77\times 10^{-15}$	$4.93\times 10^{0}$	$2.16\times 10^{-15}$	$2.35\times 10^{-15}$	$1.66\times 10^{-5}$	$1.80\times 10^{-15}$	$2.12\times 10^{1}$	$7.20\times 10^{0}$	$3.71\times 10^{-5}$	$2.37\times 10^{-4}$	$1.77\times 10^{-2}$	$1.35\times 10^{-15}$
	avg	$3.00\times 10^{0}$	$3.90\times 10^{0}$	$3.00\times 10^{0}$	$3.00\times 10^{0}$	$3.00\times 10^{0}$	$3.00\times 10^{0}$	$1.02\times 10^{1}$	$4.86\times 10^{0}$	$3.00\times 10^{0}$	$3.00\times 10^{0}$	$3.01\times 10^{0}$	$3.00\times 10^{0}$
F19	min	$-3.86\times 10^{0}$	$-3.86\times 10^{0}$	$-3.86\times 10^{0}$	$-3.86\times 10^{0}$	$-3.86\times 10^{0}$	$-3.86\times 10^{0}$	$-3.86\times 10^{0}$	$-3.84\times 10^{0}$	$-3.86\times 10^{0}$	$-3.86\times 10^{0}$	$-3.86\times 10^{0}$	$-3.86\times 10^{0}$
	std	$2.60\times 10^{-15}$	$2.65\times 10^{-15}$	$2.68\times 10^{-15}$	$2.52\times 10^{-15}$	$1.68\times 10^{-2}$	$2.70\times 10^{-15}$	$3.05\times 10^{-6}$	$3.78\times 10^{-1}$	$2.32\times 10^{-3}$	$7.81\times 10^{-3}$	$2.78\times 10^{-3}$	$2.70\times 10^{-15}$
	avg	$-3.86\times 10^{0}$	$-3.86\times 10^{0}$	$-3.86\times 10^{0}$	$-3.86\times 10^{0}$	$-3.84\times 10^{0}$	$-3.86\times 10^{0}$	$-3.86\times 10^{0}$	$-3.24\times 10^{0}$	$-3.86\times 10^{0}$	$-3.86\times 10^{0}$	$-3.86\times 10^{0}$	$-3.86\times 10^{0}$
F20	min	$-3.32\times 10^{0}$	$-3.32\times 10^{0}$	$-3.32\times 10^{0}$	$-3.32\times 10^{0}$	$-3.07\times 10^{0}$	$-3.32\times 10^{0}$	$-3.32\times 10^{0}$	$-2.51\times 10^{0}$	$-3.32\times 10^{0}$	$-3.32\times 10^{0}$	$-3.32\times 10^{0}$	$-3.32\times 10^{0}$
	std	$6.03\times 10^{-2}$	$6.05\times 10^{-2}$	$6.07\times 10^{-2}$	$4.84\times 10^{-2}$	$2.21\times 10^{-1}$	$6.39\times 10^{-2}$	$6.19\times 10^{-2}$	$4.74\times 10^{-1}$	$7.75\times 10^{-2}$	$1.17\times 10^{-1}$	$1.29\times 10^{-1}$	$5.70\times 10^{-2}$
	avg	$-3.27\times 10^{0}$	$-3.26\times 10^{0}$	$-3.26\times 10^{0}$	$-3.30\times 10^{0}$	$-2.86\times 10^{0}$	$-3.26\times 10^{0}$	$-3.25\times 10^{0}$	$-1.47\times 10^{0}$	$-3.28\times 10^{0}$	$-3.19\times 10^{0}$	$-3.10\times 10^{0}$	$-3.28\times 10^{0}$
F21	min	$-1.02\times 10^{1}$	$-1.02\times 10^{1}$	$-1.02\times 10^{1}$	$-5.06\times 10^{0}$	$-1.02\times 10^{1}$	$-1.02\times 10^{1}$	$-1.02\times 10^{1}$	$-4.83\times 10^{0}$	$-1.02\times 10^{1}$	$-1.02\times 10^{1}$	$-1.02\times 10^{1}$	$-1.02\times 10^{1}$
	std	$1.37\times 10^{0}$	$3.42\times 10^{0}$	$3.02\times 10^{0}$	$1.62\times 10^{-15}$	$5.70\times 10^{-4}$	$2.93\times 10^{0}$	$3.15\times 10^{0}$	$7.78\times 10^{-1}$	$2.82\times 10^{0}$	$2.90\times 10^{0}$	$1.72\times 10^{0}$	$1.29\times 10^{0}$
	avg	$-9.90\times 10^{0}$	$-6.89\times 10^{0}$	$-7.96\times 10^{0}$	$-5.06\times 10^{0}$	$-1.02\times 10^{1}$	$-4.73\times 10^{0}$	$-5.30\times 10^{0}$	$-1.94\times 10^{0}$	$-8.39\times 10^{0}$	$-8.03\times 10^{0}$	$-5.72\times 10^{0}$	$-9.82\times 10^{0}$
F22	min	$-1.04\times 10^{1}$	$-1.04\times 10^{1}$	$-1.04\times 10^{1}$	$-1.04\times 10^{1}$	$-1.04\times 10^{1}$	$-1.04\times 10^{1}$	$-1.04\times 10^{1}$	$-4.16\times 10^{0}$	$-1.04\times 10^{1}$	$-1.04\times 10^{1}$	$-1.04\times 10^{1}$	$-1.04\times 10^{1}$
	std	$1.39\times 10^{0}$	$3.60\times 10^{0}$	$3.16\times 10^{0}$	$1.13\times 10^{0}$	$6.53\times 10^{-4}$	$3.82\times 10^{0}$	$3.13\times 10^{0}$	$7.73\times 10^{-1}$	$1.62\times 10^{0}$	$3.14\times 10^{0}$	$1.62\times 10^{0}$	$2.85\times 10^{0}$
	avg	$-1.02\times 10^{1}$	$-7.36\times 10^{0}$	$-8.10\times 10^{0}$	$-5.38\times 10^{0}$	$-1.04\times 10^{1}$	$-6.92\times 10^{0}$	$-5.07\times 10^{0}$	$-1.98\times 10^{0}$	$-9.87\times 10^{0}$	$-7.14\times 10^{0}$	$-5.62\times 10^{0}$	$-8.87\times 10^{0}$
F23	min	$-1.05\times 10^{1}$	$-1.05\times 10^{1}$	$-1.05\times 10^{1}$	$-1.05\times 10^{1}$	$-1.05\times 10^{1}$	$-1.05\times 10^{1}$	$-1.05\times 10^{1}$	$-3.83\times 10^{0}$	$-1.05\times 10^{1}$	$-1.05\times 10^{1}$	$-1.05\times 10^{1}$	$-1.05\times 10^{1}$
	std	$2.54\times 10^{0}$	$3.49\times 10^{0}$	$3.24\times 10^{0}$	$2.75\times 10^{0}$	$1.85\times 10^{-3}$	$3.78\times 10^{0}$	$2.99\times 10^{0}$	$8.49\times 10^{-1}$	$1.09\times 10^{-3}$	$3.39\times 10^{0}$	$2.25\times 10^{0}$	$8.73\times 10^{-7}$
	avg	$-9.42\times 10^{0}$	$-8.16\times 10^{0}$	$-8.17\times 10^{0}$	$-7.83\times 10^{0}$	$-1.05\times 10^{1}$	$-6.31\times 10^{0}$	$-4.27\times 10^{0}$	$-1.92\times 10^{0}$	$-1.05\times 10^{1}$	$-6.34\times 10^{0}$	$-6.43\times 10^{0}$	$-1.05\times 10^{1}$

**Table 2:** Wilcoxon rank-sum test results comparing the performance of optimization algorithms on 23 benchmark functions.

Func.	KEO	ALA	WUTP	FATA	PSO	GOOSE	GA	GWO	WOA	DBO	APO
F1	$1.21\times 10^{-12}$	$1.21\times 10^{-12}$	$1.21\times 10^{-12}$	$1.00\times 10^{0}$	$1.21\times 10^{-12}$	$1.21\times 10^{-12}$	$1.21\times 10^{-12}$	$1.21\times 10^{-12}$	$1.21\times 10^{-12}$	$1.00\times 10^{0}$	$1.21\times 10^{-12}$
F2	$3.02\times 10^{-11}$	$3.02\times 10^{-11}$	$3.02\times 10^{-11}$	$1.21\times 10^{-12}$	$3.02\times 10^{-11}$	$3.02\times 10^{-11}$	$3.02\times 10^{-11}$	$3.02\times 10^{-11}$	$3.02\times 10^{-11}$	$1.21\times 10^{-12}$	$3.02\times 10^{-11}$
F3	$1.21\times 10^{-12}$	$1.21\times 10^{-12}$	$1.21\times 10^{-12}$	$1.00\times 10^{0}$	$1.21\times 10^{-12}$	$1.21\times 10^{-12}$	$1.21\times 10^{-12}$	$1.21\times 10^{-12}$	$1.21\times 10^{-12}$	$4.19\times 10^{-2}$	$1.21\times 10^{-12}$
F4	$3.02\times 10^{-11}$	$3.02\times 10^{-11}$	$3.02\times 10^{-11}$	$1.21\times 10^{-12}$	$3.02\times 10^{-11}$	$3.02\times 10^{-11}$	$3.02\times 10^{-11}$	$3.02\times 10^{-11}$	$3.02\times 10^{-11}$	$2.51\times 10^{-2}$	$3.02\times 10^{-11}$
F5	$3.02\times 10^{-11}$	$2.20\times 10^{-7}$	$3.02\times 10^{-11}$	$1.11\times 10^{-6}$	$3.02\times 10^{-11}$	$5.57\times 10^{-10}$	$3.02\times 10^{-11}$	$4.35\times 10^{-5}$	$3.02\times 10^{-11}$	$3.02\times 10^{-11}$	$3.02\times 10^{-11}$
F6	$3.02\times 10^{-11}$	$4.50\times 10^{-11}$	$3.02\times 10^{-11}$	$3.02\times 10^{-11}$	$3.02\times 10^{-11}$	$3.02\times 10^{-11}$	$3.02\times 10^{-11}$	$3.02\times 10^{-11}$	$3.02\times 10^{-11}$	$3.02\times 10^{-11}$	$3.02\times 10^{-11}$
F7	$3.02\times 10^{-11}$	$3.02\times 10^{-11}$	$3.02\times 10^{-11}$	$2.92\times 10^{-9}$	$3.02\times 10^{-11}$	$3.02\times 10^{-11}$	$3.02\times 10^{-11}$	$3.02\times 10^{-11}$	$3.02\times 10^{-11}$	$3.02\times 10^{-11}$	$3.02\times 10^{-11}$
F8	$1.29\times 10^{-6}$	$2.83\times 10^{-8}$	$3.02\times 10^{-11}$	$3.02\times 10^{-11}$	$1.49\times 10^{-6}$	$1.95\times 10^{-3}$	$3.02\times 10^{-11}$	$1.29\times 10^{-9}$	$4.57\times 10^{-9}$	$5.37\times 10^{-2}$	$2.02\times 10^{-8}$
F9	$1.21\times 10^{-12}$	$1.21\times 10^{-12}$	$1.21\times 10^{-12}$	$1.00\times 10^{0}$	$1.21\times 10^{-12}$	$1.21\times 10^{-12}$	$1.21\times 10^{-12}$	$1.18\times 10^{-12}$	$3.34\times 10^{-1}$	$1.00\times 10^{0}$	$1.21\times 10^{-12}$
F10	$1.21\times 10^{-12}$	$1.21\times 10^{-12}$	$1.21\times 10^{-12}$	$1.00\times 10^{0}$	$1.21\times 10^{-12}$	$1.21\times 10^{-12}$	$1.21\times 10^{-12}$	$1.15\times 10^{-12}$	$3.35\times 10^{-8}$	$1.00\times 10^{0}$	$1.21\times 10^{-12}$
F11	$1.21\times 10^{-12}$	$1.21\times 10^{-12}$	$1.21\times 10^{-12}$	$1.00\times 10^{0}$	$1.21\times 10^{-12}$	$1.21\times 10^{-12}$	$1.21\times 10^{-12}$	$2.79\times 10^{-3}$	$3.34\times 10^{-1}$	$1.00\times 10^{0}$	$1.21\times 10^{-12}$
F12	$3.02\times 10^{-11}$	$3.02\times 10^{-11}$	$3.02\times 10^{-11}$	$3.02\times 10^{-11}$	$3.02\times 10^{-11}$	$3.02\times 10^{-11}$	$3.02\times 10^{-11}$	$3.02\times 10^{-11}$	$3.02\times 10^{-11}$	$3.02\times 10^{-11}$	$3.02\times 10^{-11}$
F13	$4.62\times 10^{-10}$	$6.72\times 10^{-10}$	$1.44\times 10^{-2}$	$7.12\times 10^{-9}$	$3.02\times 10^{-11}$	$8.15\times 10^{-5}$	$3.02\times 10^{-11}$	$8.48\times 10^{-9}$	$8.48\times 10^{-9}$	$8.48\times 10^{-9}$	$5.49\times 10^{-11}$
F14	$6.14\times 10^{-1}$	$4.11\times 10^{-7}$	$5.28\times 10^{-5}$	$7.96\times 10^{-1}$	$3.02\times 10^{-1}$	$3.96\times 10^{-10}$	$1.85\times 10^{-1}$	$3.07\times 10^{-5}$	$6.02\times 10^{-3}$	$3.14\times 10^{-3}$	$1.82\times 10^{-1}$
F15	$1.70\times 10^{-10}$	$9.34\times 10^{-10}$	$2.85\times 10^{-11}$	$1.88\times 10^{-10}$	$2.76\times 10^{-10}$	$2.89\times 10^{-11}$	$2.89\times 10^{-11}$	$1.16\times 10^{-10}$	$9.51\times 10^{-11}$	$7.82\times 10^{-11}$	$3.53\times 10^{-11}$
F16	$1.13\times 10^{-1}$	$1.91\times 10^{-1}$	$1.00\times 10^{-1}$	$1.45\times 10^{-11}$	$1.26\times 10^{-4}$	$1.45\times 10^{-11}$	$1.45\times 10^{-11}$	$1.45\times 10^{-11}$	$1.45\times 10^{-11}$	$1.45\times 10^{-11}$	$6.93\times 10^{-5}$
F17	$1.00\times 10^{0}$	$1.00\times 10^{0}$	$1.00\times 10^{0}$	$1.21\times 10^{-12}$	$1.00\times 10^{0}$	$1.21\times 10^{-12}$	$1.21\times 10^{-12}$	$1.21\times 10^{-12}$	$1.21\times 10^{-12}$	$1.21\times 10^{-12}$	$2.21\times 10^{-6}$
F18	$1.84\times 10^{-1}$	$7.55\times 10^{-6}$	$1.38\times 10^{-3}$	$2.93\times 10^{-11}$	$2.91\times 10^{-7}$	$2.93\times 10^{-11}$	$2.93\times 10^{-11}$	$2.93\times 10^{-11}$	$2.93\times 10^{-11}$	$2.93\times 10^{-11}$	$5.58\times 10^{-11}$
F19	$2.04\times 10^{-1}$	$4.04\times 10^{-2}$	$1.83\times 10^{-1}$	$8.87\times 10^{-12}$	$1.25\times 10^{-2}$	$8.87\times 10^{-12}$	$8.87\times 10^{-12}$	$8.87\times 10^{-12}$	$8.87\times 10^{-12}$	$8.87\times 10^{-12}$	$1.25\times 10^{-2}$
F20	$8.34\times 10^{-1}$	$8.34\times 10^{-1}$	$7.56\times 10^{-2}$	$2.07\times 10^{-11}$	$6.56\times 10^{-1}$	$6.67\times 10^{-5}$	$2.07\times 10^{-11}$	$5.74\times 10^{-2}$	$1.06\times 10^{-5}$	$1.54\times 10^{-8}$	$3.62\times 10^{-1}$
F21	$1.78\times 10^{-3}$	$3.46\times 10^{-1}$	$9.05\times 10^{-11}$	$1.69\times 10^{-10}$	$5.02\times 10^{-6}$	$7.55\times 10^{-11}$	$1.17\times 10^{-11}$	$1.38\times 10^{-10}$	$1.25\times 10^{-10}$	$1.69\times 10^{-10}$	$7.65\times 10^{-1}$
F22	$2.63\times 10^{-2}$	$1.84\times 10^{-1}$	$9.38\times 10^{-11}$	$1.44\times 10^{-10}$	$4.04\times 10^{-2}$	$4.26\times 10^{-11}$	$8.86\times 10^{-12}$	$1.44\times 10^{-10}$	$9.65\times 10^{-11}$	$1.44\times 10^{-10}$	$3.28\times 10^{-1}$
F23	$7.51\times 10^{-1}$	$6.82\times 10^{-1}$	$4.88\times 10^{-6}$	$5.16\times 10^{-6}$	$2.21\times 10^{-1}$	$4.59\times 10^{-10}$	$7.85\times 10^{-12}$	$5.16\times 10^{-6}$	$1.58\times 10^{-7}$	$5.16\times 10^{-6}$	$2.48\times 10^{-5}$

**Table 3:** Performance comparison of optimization algorithms on CEC 2019 benchmark functions.

Func.	Res.	EKEO	KEO	ALA	WUTP	FATA	PSO	GOOSE	GA	GWO	WOA	DBO	APO
F1	min	$1.00\times 10^{0}$	$6.67\times 10^{3}$	$1.00\times 10^{0}$	$9.26\times 10^{5}$	$1.00\times 10^{0}$	$5.09\times 10^{3}$	$1.00\times 10^{0}$	$3.82\times 10^{6}$	$1.00\times 10^{0}$	$4.00\times 10^{3}$	$1.00\times 10^{0}$	$1.33\times 10^{3}$
	std	$0.00\times 10^{0}$	$4.36\times 10^{5}$	$6.57\times 10^{0}$	$6.76\times 10^{6}$	$2.06\times 10^{-7}$	$4.76\times 10^{5}$	$1.30\times 10^{9}$	$2.59\times 10^{7}$	$1.08\times 10^{5}$	$1.50\times 10^{7}$	$1.75\times 10^{-10}$	$4.91\times 10^{4}$
	avg	$1.00\times 10^{0}$	$3.07\times 10^{5}$	$2.62\times 10^{0}$	$7.34\times 10^{6}$	$1.00\times 10^{0}$	$2.54\times 10^{5}$	$9.77\times 10^{8}$	$3.50\times 10^{7}$	$4.85\times 10^{4}$	$1.28\times 10^{7}$	$1.00\times 10^{0}$	$5.38\times 10^{4}$
F2	min	$4.31\times 10^{0}$	$1.21\times 10^{2}$	$5.00\times 10^{0}$	$3.91\times 10^{3}$	$4.22\times 10^{0}$	$9.41\times 10^{1}$	$4.26\times 10^{0}$	$1.22\times 10^{3}$	$3.65\times 10^{1}$	$7.99\times 10^{1}$	$4.24\times 10^{0}$	$9.21\times 10^{1}$
	std	$1.26\times 10^{-1}$	$1.41\times 10^{2}$	$7.06\times 10^{0}$	$1.38\times 10^{3}$	$4.99\times 10^{-2}$	$5.23\times 10^{2}$	$2.00\times 10^{4}$	$2.79\times 10^{3}$	$2.40\times 10^{2}$	$4.08\times 10^{3}$	$3.42\times 10^{-1}$	$1.69\times 10^{2}$
	avg	$4.98\times 10^{0}$	$3.39\times 10^{2}$	$8.79\times 10^{0}$	$5.89\times 10^{3}$	$4.28\times 10^{0}$	$5.06\times 10^{2}$	$1.78\times 10^{4}$	$6.25\times 10^{3}$	$3.87\times 10^{2}$	$7.55\times 10^{3}$	$4.70\times 10^{0}$	$3.10\times 10^{2}$
F3	min	$1.00\times 10^{0}$	$1.41\times 10^{0}$	$1.41\times 10^{0}$	$6.47\times 10^{0}$	$1.10\times 10^{1}$	$1.41\times 10^{0}$	$1.45\times 10^{0}$	$4.56\times 10^{0}$	$1.39\times 10^{0}$	$1.69\times 10^{0}$	$1.85\times 10^{0}$	$1.75\times 10^{0}$
	std	$1.37\times 10^{-1}$	$2.63\times 10^{0}$	$2.79\times 10^{0}$	$1.29\times 10^{0}$	$4.01\times 10^{-1}$	$2.03\times 10^{0}$	$2.48\times 10^{0}$	$1.98\times 10^{0}$	$2.63\times 10^{0}$	$1.97\times 10^{0}$	$1.74\times 10^{0}$	$7.23\times 10^{-1}$
	avg	$1.38\times 10^{0}$	$5.39\times 10^{0}$	$5.34\times 10^{0}$	$8.77\times 10^{0}$	$1.26\times 10^{1}$	$2.74\times 10^{0}$	$8.92\times 10^{0}$	$8.50\times 10^{0}$	$3.48\times 10^{0}$	$5.32\times 10^{0}$	$4.89\times 10^{0}$	$2.89\times 10^{0}$
F4	min	$1.00\times 10^{0}$	$4.98\times 10^{0}$	$1.00\times 10^{0}$	$2.31\times 10^{1}$	$1.00\times 10^{0}$	$7.96\times 10^{0}$	$1.00\times 10^{0}$	$2.21\times 10^{0}$	$1.00\times 10^{0}$	$1.00\times 10^{0}$	$1.00\times 10^{0}$	$1.94\times 10^{0}$
	std	$0.00\times 10^{0}$	$5.79\times 10^{0}$	$1.82\times 10^{-1}$	$6.12\times 10^{0}$	$0.00\times 10^{0}$	$2.72\times 10^{0}$	$2.36\times 10^{1}$	$5.09\times 10^{0}$	$1.93\times 10^{0}$	$1.30\times 10^{1}$	$0.00\times 10^{0}$	$8.18\times 10^{-1}$
	avg	$1.00\times 10^{0}$	$1.35\times 10^{1}$	$1.03\times 10^{0}$	$3.53\times 10^{1}$	$1.00\times 10^{0}$	$1.22\times 10^{1}$	$4.26\times 10^{1}$	$9.43\times 10^{0}$	$1.74\times 10^{0}$	$5.82\times 10^{0}$	$1.00\times 10^{0}$	$3.62\times 10^{0}$
F5	min	$1.00\times 10^{0}$	$1.03\times 10^{0}$	$1.00\times 10^{0}$	$1.21\times 10^{0}$	$1.00\times 10^{0}$	$1.01\times 10^{0}$	$1.05\times 10^{0}$	$1.58\times 10^{0}$	$1.00\times 10^{0}$	$1.00\times 10^{0}$	$1.00\times 10^{0}$	$1.00\times 10^{0}$
	std	$0.00\times 10^{0}$	$9.08\times 10^{-2}$	$1.07\times 10^{-1}$	$1.17\times 10^{-1}$	$0.00\times 10^{0}$	$5.42\times 10^{-2}$	$3.58\times 10^{0}$	$1.47\times 10^{-1}$	$2.65\times 10^{-2}$	$1.17\times 10^{-1}$	$0.00\times 10^{0}$	$5.91\times 10^{-3}$
	avg	$1.00\times 10^{0}$	$1.14\times 10^{0}$	$1.07\times 10^{0}$	$1.46\times 10^{0}$	$1.00\times 10^{0}$	$1.10\times 10^{0}$	$3.42\times 10^{0}$	$2.00\times 10^{0}$	$1.02\times 10^{0}$	$1.09\times 10^{0}$	$1.00\times 10^{0}$	$1.00\times 10^{0}$
F6	min	$1.00\times 10^{0}$	$1.00\times 10^{0}$	$1.00\times 10^{0}$	$1.00\times 10^{0}$	$1.00\times 10^{0}$	$1.00\times 10^{0}$	$1.03\times 10^{0}$	$2.21\times 10^{0}$	$1.00\times 10^{0}$	$1.00\times 10^{0}$	$1.00\times 10^{0}$	$1.00\times 10^{0}$
	std	$0.00\times 10^{0}$	$6.15\times 10^{-1}$	$1.93\times 10^{-2}$	$2.33\times 10^{-3}$	$0.00\times 10^{0}$	$7.29\times 10^{-1}$	$4.99\times 10^{0}$	$1.93\times 10^{0}$	$0.00\times 10^{0}$	$6.57\times 10^{-16}$	$0.00\times 10^{0}$	$2.35\times 10^{-5}$
	avg	$1.00\times 10^{0}$	$1.26\times 10^{0}$	$1.03\times 10^{0}$	$1.01\times 10^{0}$	$1.00\times 10^{0}$	$1.55\times 10^{0}$	$6.21\times 10^{0}$	$6.03\times 10^{0}$	$1.00\times 10^{0}$	$1.00\times 10^{0}$	$1.00\times 10^{0}$	$1.00\times 10^{0}$
F7	min	$1.00\times 10^{0}$	$4.66\times 10^{0}$	$1.00\times 10^{0}$	$7.59\times 10^{2}$	$1.00\times 10^{0}$	$7.95\times 10^{0}$	$1.00\times 10^{0}$	$2.44\times 10^{0}$	$1.00\times 10^{0}$	$1.00\times 10^{0}$	$1.00\times 10^{0}$	$2.09\times 10^{0}$
	std	$0.00\times 10^{0}$	$2.67\times 10^{2}$	$2.08\times 10^{2}$	$2.23\times 10^{2}$	$2.23\times 10^{-2}$	$1.96\times 10^{2}$	$7.53\times 10^{2}$	$3.66\times 10^{2}$	$3.07\times 10^{1}$	$3.23\times 10^{2}$	$0.00\times 10^{0}$	$4.16\times 10^{0}$
	avg	$1.00\times 10^{0}$	$4.37\times 10^{2}$	$5.05\times 10^{1}$	$1.30\times 10^{3}$	$1.01\times 10^{0}$	$3.60\times 10^{2}$	$8.31\times 10^{2}$	$5.68\times 10^{2}$	$8.81\times 10^{0}$	$1.27\times 10^{2}$	$1.00\times 10^{0}$	$9.65\times 10^{0}$
F8	min	$1.00\times 10^{0}$	$2.03\times 10^{0}$	$1.00\times 10^{0}$	$2.89\times 10^{0}$	$1.00\times 10^{0}$	$1.44\times 10^{0}$	$1.26\times 10^{0}$	$3.91\times 10^{0}$	$1.00\times 10^{0}$	$1.00\times 10^{0}$	$1.00\times 10^{0}$	$1.28\times 10^{0}$
	std	$0.00\times 10^{0}$	$6.65\times 10^{-1}$	$1.19\times 10^{0}$	$3.51\times 10^{-1}$	$3.78\times 10^{-3}$	$6.99\times 10^{-1}$	$1.46\times 10^{0}$	$2.68\times 10^{-1}$	$4.11\times 10^{-1}$	$1.00\times 10^{0}$	$1.89\times 10^{-1}$	$1.57\times 10^{-1}$
	avg	$1.00\times 10^{0}$	$3.30\times 10^{0}$	$2.28\times 10^{0}$	$4.14\times 10^{0}$	$1.00\times 10^{0}$	$2.74\times 10^{0}$	$4.54\times 10^{0}$	$4.89\times 10^{0}$	$1.57\times 10^{0}$	$1.64\times 10^{0}$	$1.03\times 10^{0}$	$1.54\times 10^{0}$
F9	min	$1.00\times 10^{0}$	$1.07\times 10^{0}$	$1.00\times 10^{0}$	$1.12\times 10^{0}$	$1.00\times 10^{0}$	$1.09\times 10^{0}$	$1.03\times 10^{0}$	$1.13\times 10^{0}$	$1.02\times 10^{0}$	$1.11\times 10^{0}$	$1.00\times 10^{0}$	$1.04\times 10^{0}$
	std	$1.96\times 10^{-16}$	$1.08\times 10^{-1}$	$6.24\times 10^{-2}$	$5.54\times 10^{-2}$	$2.36\times 10^{-2}$	$9.70\times 10^{-2}$	$2.51\times 10^{-1}$	$5.42\times 10^{-2}$	$3.47\times 10^{-2}$	$1.19\times 10^{-1}$	$4.44\times 10^{-2}$	$2.29\times 10^{-2}$
	avg	$1.00\times 10^{0}$	$1.23\times 10^{0}$	$1.09\times 10^{0}$	$1.24\times 10^{0}$	$1.02\times 10^{0}$	$1.23\times 10^{0}$	$1.29\times 10^{0}$	$1.22\times 10^{0}$	$1.09\times 10^{0}$	$1.33\times 10^{0}$	$1.04\times 10^{0}$	$1.07\times 10^{0}$
F10	min	$1.00\times 10^{0}$	$7.62\times 10^{0}$	$1.00\times 10^{0}$	$2.10\times 10^{1}$	$1.00\times 10^{0}$	$1.00\times 10^{0}$	$1.01\times 10^{0}$	$2.13\times 10^{1}$	$1.00\times 10^{0}$	$1.00\times 10^{0}$	$1.00\times 10^{0}$	$2.10\times 10^{1}$
	std	$0.00\times 10^{0}$	$2.44\times 10^{0}$	$8.59\times 10^{0}$	$1.18\times 10^{-8}$	$0.00\times 10^{0}$	$7.97\times 10^{0}$	$8.77\times 10^{0}$	$1.03\times 10^{-1}$	$6.17\times 10^{0}$	$3.65\times 10^{0}$	$6.57\times 10^{-16}$	$1.91\times 10^{-5}$
	avg	$1.00\times 10^{0}$	$2.06\times 10^{1}$	$5.69\times 10^{0}$	$2.10\times 10^{1}$	$1.00\times 10^{0}$	$1.71\times 10^{1}$	$1.49\times 10^{1}$	$2.15\times 10^{1}$	$1.92\times 10^{1}$	$1.67\times 10^{0}$	$1.00\times 10^{0}$	$2.10\times 10^{1}$

**Table 4:** Wilcoxon rank-sum test results comparing the performance of optimization algorithms on the CEC 2019 functions.

Func.	KEO	ALA	WUTP	FATA	PSO	GOOSE	GA	GWO	WOA	DBO	APO
F1	$1.21\times 10^{-12}$	$5.85\times 10^{-9}$	$1.21\times 10^{-12}$	$1.21\times 10^{-12}$	$1.21\times 10^{-12}$	$1.21\times 10^{-12}$	$1.21\times 10^{-12}$	$1.21\times 10^{-12}$	$1.21\times 10^{-12}$	$3.34\times 10^{-1}$	$1.21\times 10^{-12}$
F2	$1.72\times 10^{-12}$	$1.72\times 10^{-12}$	$1.72\times 10^{-12}$	$3.02\times 10^{-12}$	$1.72\times 10^{-12}$	$1.29\times 10^{-2}$	$1.72\times 10^{-12}$	$1.72\times 10^{-12}$	$1.72\times 10^{-12}$	$1.80\times 10^{-9}$	$1.72\times 10^{-12}$
F3	$9.26\times 10^{-9}$	$1.41\times 10^{-9}$	$3.02\times 10^{-11}$	$3.02\times 10^{-11}$	$2.50\times 10^{-3}$	$3.69\times 10^{-11}$	$3.02\times 10^{-11}$	$2.03\times 10^{-9}$	$3.02\times 10^{-11}$	$3.02\times 10^{-11}$	$3.02\times 10^{-11}$
F4	$1.20\times 10^{-12}$	$1.21\times 10^{-12}$	$1.21\times 10^{-12}$	$1.00\times 10^{0}$	$1.20\times 10^{-12}$	$1.21\times 10^{-12}$	$1.21\times 10^{-12}$	$2.16\times 10^{-2}$	$2.16\times 10^{-2}$	$1.00\times 10^{0}$	$1.21\times 10^{-12}$
F5	$1.21\times 10^{-12}$	$1.21\times 10^{-12}$	$1.21\times 10^{-12}$	$1.00\times 10^{0}$	$1.21\times 10^{-12}$	$1.21\times 10^{-12}$	$1.21\times 10^{-12}$	$2.21\times 10^{-6}$	$2.93\times 10^{-5}$	$1.00\times 10^{0}$	$1.21\times 10^{-12}$
F6	$1.21\times 10^{-12}$	$1.21\times 10^{-12}$	$1.21\times 10^{-12}$	$1.00\times 10^{0}$	$1.21\times 10^{-12}$	$1.21\times 10^{-12}$	$1.21\times 10^{-12}$	$1.00\times 10^{0}$	$3.34\times 10^{-1}$	$1.00\times 10^{0}$	$1.21\times 10^{-12}$
F7	$1.21\times 10^{-12}$	$1.21\times 10^{-12}$	$1.21\times 10^{-12}$	$1.10\times 10^{-2}$	$1.21\times 10^{-12}$	$1.21\times 10^{-12}$	$1.21\times 10^{-12}$	$1.20\times 10^{-12}$	$1.24\times 10^{-5}$	$1.00\times 10^{0}$	$1.21\times 10^{-12}$
F8	$1.21\times 10^{-12}$	$1.21\times 10^{-12}$	$1.21\times 10^{-12}$	$1.46\times 10^{-4}$	$1.21\times 10^{-12}$	$1.21\times 10^{-12}$	$1.21\times 10^{-12}$	$4.57\times 10^{-12}$	$6.62\times 10^{-4}$	$3.34\times 10^{-1}$	$1.21\times 10^{-12}$
F9	$1.17\times 10^{-11}$	$1.17\times 10^{-11}$	$1.17\times 10^{-11}$	$4.58\times 10^{-8}$	$1.17\times 10^{-11}$	$1.17\times 10^{-11}$	$1.17\times 10^{-11}$	$1.17\times 10^{-11}$	$1.17\times 10^{-11}$	$1.22\times 10^{-9}$	$1.17\times 10^{-11}$
F10	$1.60\times 10^{-13}$	$1.21\times 10^{-12}$	$4.16\times 10^{-14}$	$1.00\times 10^{0}$	$1.21\times 10^{-12}$	$1.21\times 10^{-12}$	$1.21\times 10^{-12}$	$1.21\times 10^{-12}$	$3.67\times 10^{-10}$	$3.34\times 10^{-1}$	$8.31\times 10^{-13}$

**Table 5:** Optimization parameter results of the step-cone pulley design problem.

Res.	EKEO	KEO	ALA	WUTP	FATA	PSO	GOOSE	GA	GWO	WOA	DBO	APO
$l_{1}$	38.56	40.04	39.94	40.92	40.56	39.59	38.56	39.42	40.89	39.64	40.86	40.91
$l_{2}$	53.06	55.10	54.96	56.31	56.72	54.48	53.07	54.25	56.26	54.55	56.23	56.30
$l_{3}$	70.74	73.46	73.27	75.07	75.93	72.63	70.75	72.32	75.00	72.73	75.16	75.06
$l_{4}$	84.82	88.08	87.85	90.00	90.00	87.08	84.83	86.71	89.91	87.20	90.00	89.99
ω	90.00	86.41	87.09	84.54	90.00	90.00	89.93	88.43	87.20	87.20	90.00	84.53
$f(\bar{x})$	16.21	16.79	16.84	17.15	$3.07\times 10^{10}$	17.09	$3.20\times 10^{3}$	$4.60\times 10^{4}$	$8.35\times 10^{6}$	41.16	$3.63\times 10^{8}$	17.35
rank	1	2	3	5	12	4	8	9	10	7	11	6

**Table 6:** Indicator statistical results of the step-cone pulley design problem.

Alg.	EKEO	KEO	ALA	WUTP	FATA	PSO	GOOSE	GA	GWO	WOA	DBO	APO
min	16.21	16.79	16.84	17.15	$3.07\times 10^{10}$	17.09	$3.20\times 10^{3}$	$4.60\times 10^{4}$	$8.35\times 10^{6}$	41.16	$3.63\times 10^{8}$	17.35
worst	18.24	17.14	232.30	$7.04\times 10^{4}$	$1.84\times 10^{11}$	$8.40\times 10^{11}$	$8.83\times 10^{12}$	$1.02\times 10^{10}$	$1.63\times 10^{8}$	$1.01\times 10^{7}$	$4.96\times 10^{11}$	222.86
std	0.64	0.11	75.81	$2.22\times 10^{4}$	$3.87\times 10^{10}$	$2.66\times 10^{11}$	$2.78\times 10^{12}$	$3.17\times 10^{9}$	$4.81\times 10^{7}$	$3.19\times 10^{6}$	$1.92\times 10^{11}$	94.68
avg	17.25	17.04	64.47	7146.65	$1.08\times 10^{11}$	$8.40\times 10^{10}$	$9.18\times 10^{11}$	$1.25\times 10^{9}$	$7.22\times 10^{7}$	$1.03\times 10^{6}$	$1.37\times 10^{11}$	103.20
median	17.33	17.09	30.07	21.85	$1.06\times 10^{11}$	18.18	$2.11\times 10^{4}$	$1.76\times 10^{8}$	$6.53\times 10^{7}$	$1.91\times 10^{4}$	$6.96\times 10^{10}$	41.20

**Table 7:** Optimization parameter results of the gear train design problem.

Res.	EKEO	KEO	ALA	WUTP	FATA	PSO	GOOSE	GA	GWO	WOA	DBO	APO
$G_{a}$	58.28	43.82	57.09	60.00	60.00	47.16	37.37	59.06	52.27	43.25	26.96	51.70
$G_{b}$	12.02	28.63	14.74	12.00	43.28	15.21	22.56	16.45	13.94	16.57	13.10	31.29
$G_{d}$	12.00	12.00	12.00	12.00	12.00	13.57	12.34	29.24	29.93	16.57	14.29	12.47
$G_{f}$	17.15	54.34	21.48	16.63	60.00	30.32	51.61	56.43	55.31	43.98	48.12	52.31
$f(\bar{x})$	0.00	0.00	0.00	$7.39\times 10^{-23}$	$2.12\times 10^{-11}$	0.00	$1.10\times 10^{-16}$	$8.34\times 10^{-18}$	$5.04\times 10^{-14}$	0.00	$1.32\times 10^{-12}$	$7.58\times 10^{-18}$
rank	1	4	5	6	12	2	9	8	10	3	11	7

**Table 8:** Indicator statistical results of the gear train design problem.

Alg.	EKEO	KEO	ALA	WUTP	FATA	PSO	GOOSE	GA	GWO	WOA	DBO	APO
min	0.00	0.00	0.00	$7.39\times 10^{-23}$	$2.12\times 10^{-11}$	0.00	$1.10\times 10^{-16}$	$8.34\times 10^{-18}$	$5.04\times 10^{-14}$	0.00	$1.32\times 10^{-12}$	$7.58\times 10^{-18}$
worst	0.00	$9.68\times 10^{-20}$	$6.63\times 10^{-15}$	$2.00\times 10^{-9}$	$2.41\times 10^{-8}$	$1.70\times 10^{-30}$	$3.66\times 10^{-14}$	$6.58\times 10^{-15}$	$1.12\times 10^{-10}$	$2.71\times 10^{-27}$	$6.09\times 10^{-10}$	$3.82\times 10^{-14}$
std	0.00	$3.06\times 10^{-20}$	$2.19\times 10^{-15}$	$6.75\times 10^{-10}$	$7.51\times 10^{-9}$	$5.34\times 10^{-31}$	$1.13\times 10^{-14}$	$2.06\times 10^{-15}$	$3.37\times 10^{-11}$	$8.59\times 10^{-28}$	$1.93\times 10^{-10}$	$1.40\times 10^{-14}$
avg	0.00	$9.68\times 10^{-21}$	$1.05\times 10^{-15}$	$3.79\times 10^{-10}$	$3.33\times 10^{-9}$	$1.83\times 10^{-31}$	$9.74\times 10^{-15}$	$8.58\times 10^{-16}$	$1.67\times 10^{-11}$	$3.37\times 10^{-28}$	$1.53\times 10^{-10}$	$1.01\times 10^{-14}$
median	0.00	0.00	$5.99\times 10^{-20}$	$2.81\times 10^{-11}$	$2.99\times 10^{-10}$	$3.85\times 10^{-34}$	$6.67\times 10^{-15}$	$3.93\times 10^{-17}$	$7.01\times 10^{-12}$	0.00	$8.22\times 10^{-11}$	$5.44\times 10^{-16}$

**Table 9:** Optimization parameter results of the tubular column design problem.

Res.	EKEO	KEO	ALA	WUTP	FATA	PSO	GOOSE	GA	GWO	WOA	DBO	APO
$x_{1}$	6.54	6.54	6.54	6.54	6.57	6.54	6.54	6.55	6.54	6.51	6.54	6.54
$x_{2}$	0.21	0.21	0.21	0.21	0.21	0.21	0.21	0.21	0.21	0.21	0.21	0.21
$f(\bar{x})$	26.49	26.49	26.49	26.49	26.58	26.49	26.49	26.61	26.49	26.55	26.50	26.49
rank	1	2	3	6	11	4	7	12	8	10	9	5

**Table 10:** Indicator statistical results of the tubular column design problem.

Alg.	EKEO	KEO	ALA	WUTP	FATA	PSO	GOOSE	GA	GWO	WOA	DBO	APO
min	26.49	26.49	26.49	26.49	26.58	26.49	26.49	26.61	26.49	26.55	26.50	26.49
worst	26.49	26.49	26.49	27.06	27.08	26.49	26.67	27.09	26.50	27.06	27.06	26.49
std	0.00	0.00	0.00	0.27	0.18	0.00	0.07	0.14	0.004	0.20	0.21	0.00
avg	26.49	26.49	26.49	26.66	26.78	26.49	26.52	26.79	26.49	26.95	26.83	26.49
median	26.49	26.49	26.49	26.49	26.71	26.49	26.49	26.77	26.49	27.06	26.84	26.49

**Table 11:** Optimization parameter results of the pressure vessel design problem.

Res.	EKEO	KEO	ALA	WUTP	FATA	PSO	GOOSE	GA	GWO	WOA	DBO	APO
$x_{1}$	3.43	3.46	3.50	3.56	3.92	3.55	9.49	4.37	3.55	5.54	3.62	3.53
$x_{2}$	2.03	1.82	1.77	1.91	2.17	2.00	2.76	2.35	1.83	2.69	2.00	1.99
$x_{3}$	11.39	11.39	11.39	11.39	11.27	11.39	16.01	13.61	11.39	17.64	11.18	11.35
$x_{4}$	47.78	47.78	47.78	47.78	54.10	47.78	10.50	28.62	47.81	2.70	50.38	48.21
$f(\bar{x})$	$6.06\times 10^{3}$	$6.06\times 10^{3}$	$6.06\times 10^{3}$	$6.06\times 10^{3}$	$7.20\times 10^{3}$	$6.06\times 10^{3}$	$1.32\times 10^{4}$	$7.18\times 10^{3}$	$6.06\times 10^{3}$	$7.54\times 10^{3}$	$6.15\times 10^{3}$	$6.08\times 10^{3}$
rank	1	2	3	6	11	4	7	12	8	10	9	5

**Table 12:** Indicator statistical results of the pressure vessel design problem.

Alg.	EKEO	KEO	ALA	WUTP	FATA	PSO	GOOSE	GA	GWO	WOA	DBO	APO
min	$6.06\times 10^{3}$	$6.06\times 10^{3}$	$6.06\times 10^{3}$	$6.06\times 10^{3}$	$7.20\times 10^{3}$	$6.06\times 10^{3}$	$1.32\times 10^{4}$	$7.18\times 10^{3}$	$6.06\times 10^{3}$	$7.54\times 10^{3}$	$6.15\times 10^{3}$	$6.08\times 10^{3}$
worst	$6.69\times 10^{3}$	$7.54\times 10^{3}$	$7.54\times 10^{3}$	$7.33\times 10^{3}$	$9.69\times 10^{3}$	$6.77\times 10^{3}$	$4.06\times 10^{4}$	$8.66\times 10^{3}$	$6.79\times 10^{3}$	$2.02\times 10^{4}$	$7.43\times 10^{3}$	$6.82\times 10^{3}$
std	$2.21\times 10^{2}$	$5.82\times 10^{2}$	$6.03\times 10^{2}$	$4.36\times 10^{2}$	$9.18\times 10^{2}$	$2.17\times 10^{2}$	$9.12\times 10^{3}$	$4.45\times 10^{2}$	$2.96\times 10^{2}$	$3.90\times 10^{3}$	$4.84\times 10^{2}$	$2.76\times 10^{2}$
avg	$6.24\times 10^{3}$	$6.72\times 10^{3}$	$6.50\times 10^{3}$	$6.31\times 10^{3}$	$8.35\times 10^{3}$	$6.33\times 10^{3}$	$2.98\times 10^{4}$	$8.00\times 10^{3}$	$6.24\times 10^{3}$	$1.08\times 10^{4}$	$6.75\times 10^{3}$	$6.35\times 10^{3}$
median	$6.09\times 10^{3}$	$6.60\times 10^{3}$	$6.22\times 10^{3}$	$6.09\times 10^{3}$	$8.36\times 10^{3}$	$6.37\times 10^{3}$	$3.23\times 10^{4}$	$8.00\times 10^{3}$	$6.07\times 10^{3}$	$9.11\times 10^{3}$	$6.63\times 10^{3}$	$6.37\times 10^{3}$

## Data Availability

The data presented in this study are available on request from the corresponding author. The data are not publicly available due to privacy.

## Appendix A. Mathematical Definitions of the 23 Benchmark Functions

To make it easier for readers to understand the functions used in this paper, this appendix lists the mathematical expressions, search spaces, and optimal values of all 23 benchmark functions. These functions fall into four categories: unimodal functions, basic multimodal functions, mixed functions and composite functions. All functions are minimization problems and the global minimum is known.

**Table A1:** The 23 benchmark functions.

No.	Function Definition	Range	$f_{\min}$
F1	$f_{1}\left(x\right)=\sum_{i=1}^{D}x_{i}^{2}$	[−100,100]	0
F2	$f_{2}\left(x\right)=\sum_{i=1}^{D}|x_{i}|+\prod_{i=1}^{D}\left|x_{i}\right|$	[−10,10]	0
F3	$f_{3}\left(x\right)=\sum_{i=1}^{D}{\left(\sum_{j=1}^{i}x_{j}\right)}^{2}$	[−100,100]	0
F4	$f_{4}\left(x\right)=\max_{i}\left\{\right|x_{i}\left|\right\}$	[−100,100]	0
F5	$\begin{array}{cc} f_{5}\left(x\right)= & \sum_{i=1}^{D-1}\left[100{\left(x_{i+1}-x_{i}^{2}\right)}^{2}+{\left(x_{i}-1\right)}^{2}\right] \end{array}$	[−30,30]	0
F6	$f_{6}\left(x\right)=\sum_{i=1}^{D}{\left(x_{i}+0.5\right)}^{2}$	[−100,100]	0
F7	$f_{7}\left(x\right)=\sum_{i=1}^{D}ix_{i}^{2}+\mathrm{rand}\left[0,1\right]$	[−1.28,1.28]	0
F8	$\begin{array}{cc} f_{8}\left(x\right)= & \sum_{i=1}^{D}x_{i}^{2}\times \sin\left(\sqrt{|x_{i}|}\right) \end{array}$	[−500,500]	−418.98D
F9	$\begin{array}{cc} f_{9}\left(x\right)= & \sum_{i=1}^{D}\left[x_{i}^{2}-10\cos\left(2\pi x_{i}\right)+10\right] \end{array}$	[−5.12,5.12]	0
F10	$\begin{array}{cc} f_{10}\left(x\right)= & -20\exp\left(-0.2\sqrt{\frac{1}{D}\sum_{i=1}^{D}x_{i}^{2}}\right)-\exp\left(\frac{1}{D}\sum_{i=1}^{D}\cos\left(2\pi x_{i}\right)\right)+20+e \end{array}$	[−32,32]	0
F11	$\begin{array}{cc} f_{11}\left(x\right)= & \frac{1}{4000}\sum_{i=1}^{D}x_{i}^{2}-\prod_{i=1}^{D}\cos\left(\frac{x_{i}}{\sqrt{i}}\right)+1 \end{array}$	[−600,600]	0
F12	$\begin{array}{cc} f_{12}\left(x\right)= & \frac{\pi }{D}\left[10\sin^{2}\left(\pi y_{1}\right)+\sum_{i=1}^{D-1}{\left(y_{i}-1\right)}^{2}\left(1+10\sin^{2}\left(\pi y_{i+1}\right)\right)+{\left(y_{D}-1\right)}^{2}\right]+\sum_{i=1}^{D}u\left(x_{i}\right) \end{array}$	[−50,50]	0

**Table A2:** The 23 benchmark functions.

No.	Function Definition	Range	$f_{\min}$
F13	$\begin{array}{cc} f_{13}\left(x\right)= & 0.1\left[\sin^{2}\left(3\pi x_{1}\right)+\sum_{i=1}^{D-1}{\left(x_{i}-1\right)}^{2}\left(1+\sin^{2}\left(3\pi x_{i+1}\right)\right)+{\left(x_{D}-1\right)}^{2}\left(1+\sin^{2}\left(2\pi x_{D}\right)\right)\right]+\sum_{i=1}^{D}u\left(x_{i},5\right) \end{array}$	[−50,50]	0
F14	$\begin{array}{cc} f_{14}\left(x\right)= & {\left(\frac{1}{500}+\sum_{j=1}^{25}\frac{1}{j+\sum_{i=1}^{2}{\left(x_{i}-a_{ij}\right)}^{6}}\right)}^{-1} \end{array}$	[−65,65]	1
F15	$\begin{array}{cc} f_{15}\left(x\right)= & \sum_{i=1}^{11}{\left[a_{i}-\frac{x_{1}\left(b_{i}^{2}+b_{i}x_{2}\right)}{b_{i}^{2}+b_{i}x_{3}+x_{4}}\right]}^{2} \end{array}$	[−5,5]	0.1484
F16	$\begin{array}{cc} f_{16}\left(x\right)= & 4x_{1}^{2}-2.1x_{1}^{4}+\frac{1}{3}x_{1}^{6}+x_{1}x_{2}-4x_{2}^{2}+4x_{2}^{4} \end{array}$	[−5,5]	−1.0316
F17	$\begin{array}{cc} f_{17}\left(x\right)= & {\left(x_{2}-\frac{5.1}{4\pi^{2}}x_{1}^{2}+\frac{5}{\pi }x_{1}-6\right)}^{2}+10\left(1-\frac{1}{8\pi }\right)\cos x_{1}+10 \end{array}$	[−5,5]	0.3979
F18	$\begin{array}{cc} f_{18}\left(x\right)= & \left[1+{\left(x_{1}+x_{2}+1\right)}^{2}\times \left(19-14x_{1}+3x_{1}^{2}-14x_{2}+6x_{1}x_{2}+3x_{2}^{2}\right)\right]\times \left[30+{\left(2x_{1}-3x_{2}\right)}^{2}\times \left(18-32x_{1}+12x_{1}^{2}+48x_{2}-36x_{1}x_{2}+27x_{2}^{2}\right)\right] \end{array}$	[−2,2]	3
F19	$\begin{array}{cc} f_{19}\left(x\right)= & -\sum_{i=1}^{4}c_{i}\exp\left(-\sum_{j=1}^{3}a_{ij}{\left(x_{j}-p_{ij}\right)}^{2}\right) \end{array}$	[0,1]	−3
F20	$\begin{array}{cc} f_{20}\left(x\right)= & -\sum_{i=1}^{4}c_{i}\exp\left(-\sum_{j=1}^{6}a_{ij}{\left(x_{j}-p_{ij}\right)}^{2}\right) \end{array}$	[0,1]	−3
F21	$\begin{array}{cc} f_{21}\left(x\right)= & -\sum_{i=1}^{5}{\left[\left(X-a_{i}\right){\left(X-a_{i}\right)}^{T}+c_{i}\right]}^{-1} \end{array}$	[0,10]	−1
F22	$\begin{array}{cc} f_{22}\left(x\right)= & -\sum_{i=1}^{7}{\left[\left(X-a_{i}\right){\left(X-a_{i}\right)}^{T}+c_{i}\right]}^{-1} \end{array}$	[0,10]	−1
F23	$\begin{array}{cc} f_{23}\left(x\right)= & -\sum_{i=1}^{10}{\left[\left(X-a_{i}\right){\left(X-a_{i}\right)}^{T}+c_{i}\right]}^{-1} \end{array}$	[0,10]	−1
